# A Web-Based Positive Psychological Intervention to Improve Blood Pressure Control in Spanish-Speaking Hispanic/Latino Adults With Uncontrolled Hypertension: Protocol and Design for the ¡Alégrate! Randomized Controlled Trial

**DOI:** 10.2196/17721

**Published:** 2020-08-04

**Authors:** Rosalba Hernandez, Michael Cohn, Alison Hernandez, Martha Daviglus, Lizet Martinez, Angela Martinez, Itzel Martinez, Ramon Durazo-Arvizu, Judith Moskowitz

**Affiliations:** 1 School of Social Work University of Illinois at Urbana-Champaign Urbana, IL United States; 2 Osher Center for Integrative Medicine University of California, San Francisco San Francisco, CA United States; 3 Department of Medical Social Sciences Northwestern University Feinberg School of Medicine Chicago, IL United States; 4 Institute for Minority Health Research University of Illinois at Chicago College of Medicine Chicago, IL United States; 5 Department of Psychology University of Illinois at Chicago Chicago, IL United States; 6 Stritch School of Medicine Loyola University Chicago, IL United States

**Keywords:** positive psychology, hypertension, blood pressure, emotions, telemedicine, happiness, culture, Hispanic Americans

## Abstract

**Background:**

Growing evidence links psychological well-being and resilience with superior cardiac health, but there remains a critical scientific gap about whether (or how) interventions that aim to cultivate psychological well-being reduce cardiac risk. Hispanic/Latino people in the United States have high cardiovascular disease risk and poorly controlled blood pressure (BP) compared with their peers of European ancestry, and they represent a population in need of new and innovative therapeutic approaches. As such, a focused intervention to boost psychological well-being holds promise as a novel therapeutic target for hypertension in Hispanic/Latino adults; to date, however, no research has explored whether a causal link is evident.

**Objective:**

The aim of this paper is to detail the protocol for the *¡Alégrate! (Be Happy!)* intervention, a Phase II randomized controlled trial testing initial efficacy in improving BP of a web-based positive psychological intervention designed to boost psychological well-being in Spanish-speaking Hispanic/Latino people with hypertension.

**Methods:**

A total of 70 Hispanic/Latino people aged ≥18 years, fluent in Spanish, and with elevated BP (≥140/90 mm Hg) will be recruited in person from a single Federally Qualified Health Center in Chicago. Enrollees will be randomly assigned to 1 of 2 trial arms: (1) web-based positive psychological intervention or (2) an active control condition (eg, 3 times weekly emotion reporting). Our 5-week Spanish-language *¡Alégrate!* intervention is web-based and delivers curricular content via didactic instruction, journaling, and assigned at-home practice—all accessed via our website using investigator-purchased tablet computers, with a unique username and password assigned to each enrollee. Targeted skills include noting daily positive events, positive reappraisal of stressful events, effective expression of gratitude, performing acts of kindness, and regular practice of mindfulness and meditation. The primary outcome is improvement in BP, both sitting values and 24-hour ambulatory readings, as measured at baseline and 5 and 12 weeks from baseline. Secondary outcomes include psychological well-being, engagement in healthy behaviors, and circulating levels of inflammatory markers. The outcomes of interest are collected by trained research staff through in-person interviews using the REDCap software.

**Results:**

Activities of the *¡Alégrate!* intervention were funded in August 2017, and data collection is ongoing. We expect to submit trial results for peer-reviewed publications in 2021, soon after recruitment has been concluded and statistical analyses are finalized.

**Conclusions:**

Findings will provide evidence on whether interventions to boost psychological well-being and resilience have downstream effects on BP control and cardiovascular health, particularly as they are deployed in the Spanish language with cultural tailoring and via a web-based platform. If effective, we will have an easily disseminatable application that can positively impact well-being profiles and BP control in Hispanic/Latino people, with the possibility of addressing health disparities of this US racial/ethnic minority group.

**Trial Registration:**

ClinicalTrials.gov NCT03892057; https://clinicaltrials.gov/ct2/show/NCT03892057

**International Registered Report Identifier (IRRID):**

PRR1-10.2196/17721

## Introduction

One in every 3 adults in the United States is classified as hypertensive and only around half maintain blood pressure (BP) under control [1-3]. Cardiovascular disease (CVD) is the leading cause of death in the United States, and hypertension exhibits the highest attributable risk for CVD morbidity and mortality [4]. Mounting evidence suggests that positive psychological well-being (hereafter, psychological well-being)—which includes positively valenced feelings or cognitive appraisals such as happiness, optimism, and purpose in life—is independently associated with favorable cardiac health, for example, reduced risk for incident hypertension, better lipid profiles, reduced inflammatory markers, and reduced odds of incident heart disease and cardiac-related mortality [5-7]. Nevertheless, while multiple studies link psychological well-being with better cardiac health [6-8], there is a need to investigate the most effective approaches to cultivate psychological well-being in an effort to reduce cardiac risk.

While scholars define psychological well-being in various ways [9,10], in the field of cardiovascular epidemiology, it encompasses positively valenced feelings or cognitive appraisals that individuals use to evaluate their lives favorably [7,8]. As such, 2 theoretical perspectives inform the characterization of psychological well-being: the hedonic approach, which focuses on pursuit and attainment of pleasure and happiness [11], and the eudaimonic approach, which defines well-being as the ability to practice meaningful life pursuits and striving to realize one’s best self [11]. Other domains, which are less easily classified as hedonic versus eudaimonic (eg, optimism, emotional vitality), have consistently predicted cardiovascular outcomes. Common measures of psychological well-being include purpose in life, personal growth, self-acceptance, environmental mastery, autonomy, happiness, satisfaction in life, positive affect, optimism (and hope), and emotional vitality.

The Hispanic/Latino population in the United States is a racial/ethnic group that may benefit from novel treatment focused on improving psychological well-being [12]. There are 57 million [13] Hispanic/Latino people in the United States, and they represent the second fastest growing ethnic/racial minority group [14,15]. Hispanic/Latino people exhibit a disproportionately higher burden of cardiovascular disease risk factors [3,16]. In addition, the Hispanic/Latino population has a higher incidence rate for CVD-related comorbidities (eg, chronic kidney disease) when compared with non-Hispanic populations, with 30% mortality attributable to CVD and its sequelae [14,17,18]. Additional research is needed, however, to explore strategies that boost psychological well-being specifically in Hispanic/Latino adults and whether this leads to better heart health and BP management. To date, pilot trials highlight that Hispanic/Latino adults find interventions targeting psychological well-being both enjoyable and beneficial [19,20].

Observational evidence links psychological well-being with better overall health, more healthy coping tendencies, improved quality of life, and healthy longevity [8,21]. Psychological well-being focuses on positive thoughts and feelings at the individual level, which may be key in promoting healthy behaviors in hypertension management [8]. Evidence suggests that psychological well-being is related to healthier BP profiles and plays a protective role in disease incidence [7,22]. In over 1000 healthy non-Hispanic adults, a large prospective study found that higher levels of psychological well-being were associated with a decreased likelihood of incident hypertension at a one-year follow-up [23]. In 126 British civil servants, aggregate baseline levels of happiness were negatively associated with 3-year measures of systolic BP, independent of known confounders.

Approximately 22% [15] of Hispanic/Latino people have hypertension, and they tend to display lower compliance with treatment recommendations when compared with their non-Hispanic peers [15,17]. Adherence to antihypertensive treatment and effective BP control in Hispanic/Latino people was approximately 58% and 35%, respectively, compared with whites (71% and 48%, respectively) and blacks (71% and 43%, respectively) [24]. There are no specific clinical CVD guidelines addressing the unique characteristics of Hispanic/Latino people, which highlights the need for novel and more effective disease management efforts for this racial/ethnic group [14].

The use of web-based interventions in different clinical populations has grown significantly [25]. Web-based interventions tend to be less costly than face-to-face designs, offering flexibility and greater access [26]. Internet use has also grown substantially among Hispanic/Latino adults, with 80% reporting use of the internet [26]. A majority of Hispanic/Latino people reported having a computer (78%) and reported owning a smartphone (71%) [27]. Web-based interventions have the potential for broad dissemination and may help eliminate common barriers to participation (eg, transportation, scheduling conflict, and lack of child care) [28]. Recent studies have used web-based interventions to promote psychological well-being in different patient populations [25]. For example, web-based delivery formats were found to be effective in the management of symptoms of depression, anxiety, and stress [29-33] in healthy and clinical populations. However, most of these studies have been conducted on non-Hispanic white samples. There is a need for better representation of underserved, racial/ethnic minorities in web-based psychological well-being interventions.

Empirical evidence suggests that effect sizes for web-based therapies are similar to those deployed using traditional face-to-face approaches, with high patient satisfaction reported across these high-tech platforms. Few evidence-based programs, however, have been developed specifically for and deployed with Hispanic/Latino adults using web-based platforms. Schueller et al [34] identified only 6 studies that deployed digital health technologies in Hispanic/Latino populations. Given the paucity of evidence, testing for feasibility is imperative to document whether insurmountable barriers are evident, for example, limited internet accessibility and low technology literacy, among others. For instance, in past years, Hispanic/Latino adults have reported low Wi-Fi accessibility when compared with their peers of European ancestry, but important strides have been made in recent years with 81% of Hispanic/Latino adults now reporting internet access—although, usually in the form of mobile-only internet access versus at-home networks. Nonetheless, with increased accessibility, web-based platforms can become viable avenues because of their low cost and potential for broad dissemination, particularly among marginalized, underserved, and minority populations that encounter multiple barriers in accessing care, such as lack of health insurance or shortage of Spanish-speaking clinicians. Indeed, the use of technology represents a promising opportunity as Hispanic/Latino people express high interest (ie, ~86%) in engaging health-related phone apps and related technologies [34].

*¡Alégrate! (Be Happy!)* is a Phase II randomized controlled trial (RCT) created to address these limitations by testing the initial efficacy of a Spanish language web-based intervention. The RCT intends to boost psychological well-being in Spanish-speaking Hispanic/Latino adults with hypertension by examining changes in BP, psychological well-being, healthy behavior adherence, and circulating serum inflammation. This web-based intervention is built on activities that target psychological well-being by promoting optimism, gratitude, and positive affect directly through activities such as recalling positive life events, identifying and employing personal strengths, and engaging in acts of kindness, among others. We hypothesized that compared with participants in the control condition, those in the active intervention group will show greater improvements in BP, higher scores for psychological well-being, greater engagement in healthy behaviors, and lower levels of inflammatory markers at follow-up. The aim of this paper was to detail the procedures of the ¡Alégrate! Phase II trial: Focus on the design and protocol of the web-based intervention.

## Methods

### Overview and Study Design

This study, known as *¡Alégrate! (Be Happy!)*, is an RCT testing the initial efficacy of a 5-week web-based positive psychological intervention in Spanish-speaking Hispanic/Latino adults with hypertension. As such, the current Phase II pilot trial implements a parallel group design with a 1:1 allocation ratio, with 2 fixed factors: (1) sex (male and female) and (2) antihypertensive medication use (yes and no). Participants assigned to the treatment condition will be asked to visit the *¡Alégrate!* study website over a 5-week period in which they learn skills known to boost positive emotion and overall psychological well-being, while participants in the active control condition engage in emotion reporting 3 times weekly. Participants assigned to the active control condition will gain access to the web-based curriculum at the end of the 12-week assessment period, after all survey and clinical data has been collected.

### Participant Eligibility

Spanish-speaking Hispanic/Latino adults are eligible to enroll in the *¡Alégrate!* trial if they meet the following criteria: (1) Hispanic/Latino heritage based on self-report; (2) aged 18 years or older; (3) fluent in Spanish, that is, ability to read, speak, and write Spanish; (4) elevated sitting BP (≥140/90 mm Hg); and (5) basic technological literacy with the ability to access the internet at home or in a public setting. The exclusion criteria were as follows: (1) cognitive impairment denoting dementia [35], (2) severely reduced life expectancy (eg, self-reported diagnosis of metastatic cancer, congestive heart failure, or end-stage kidney disease), and (3) severe psychopathology including active psychosis or untreated bipolar disorder. The Institutional Review Board at the University of Illinois at Urbana-Champaign (UIUC) approved the *¡Alégrate!* trial, and all subjects provided written informed consent.

### Study Procedures

#### Recruitment

Participants were recruited from a single Federally Qualified Health Center (FQHC) located in Chicago and situated in a neighborhood with a high density of Hispanic/Latino residents. The attending physician and clinical staff (eg, registered nurse, medical assistant) identify potentially eligible patients and subsequently refer them to *¡Alégrate!* research staff who then follow-up via phone or in person; participant information is stored in a secure web-based portal. During phone or in-person exchanges, the staff inform participants that the goal of the study is to explore whether an intervention intended to boost positive emotions results in better cardiac-related health. Only initial eligibility is determined via phone, with an in-person visit scheduled to establish full eligibility based on sitting B*P* values. Research staff will also approach patients in the waiting area to provide information about the research study and offer an invitation to receive a no-cost BP screening; research staff will collect contact information of those expressing interest to determine full eligibility. The consent form and study materials outline the design of the study and inform participants of random assignment into 1 of 2 groups, either the group where *they learn skills known* to *boost mood and heighten positive emotions* or the group where *they would report experiences of positive or negative emotions on a thrice weekly basis*. It is communicated that all curricular instruction occurs online by visiting our *¡Alégrate!* website. In addition, the staff inform participants that 3 in-person clinical visits are required, each 2 to 3 hours in length, where staff collect survey and clinical data including anthropometric information, blood spots, and 24-hour BP monitoring; home visits are also offered for data collection to increase rates of retention. Participants are informed that they can keep the investigator-purchased tablet computer at the end of the study as compensation for their participation and time invested [36]. To reduce the risk of bias, enrollees in both the treatment and control arms keep the tablet computer at the end of study participation, that is, immediately after providing follow-up survey and clinical data at 12 weeks postbaseline. Participants expressing interest in enrolling in the trial who are screened for full eligibility and ultimately qualify undergo a face-to-face consent process and complete the survey and clinical exams before randomization.

#### Consent, Assessments, and Randomization

**Figure 1:**
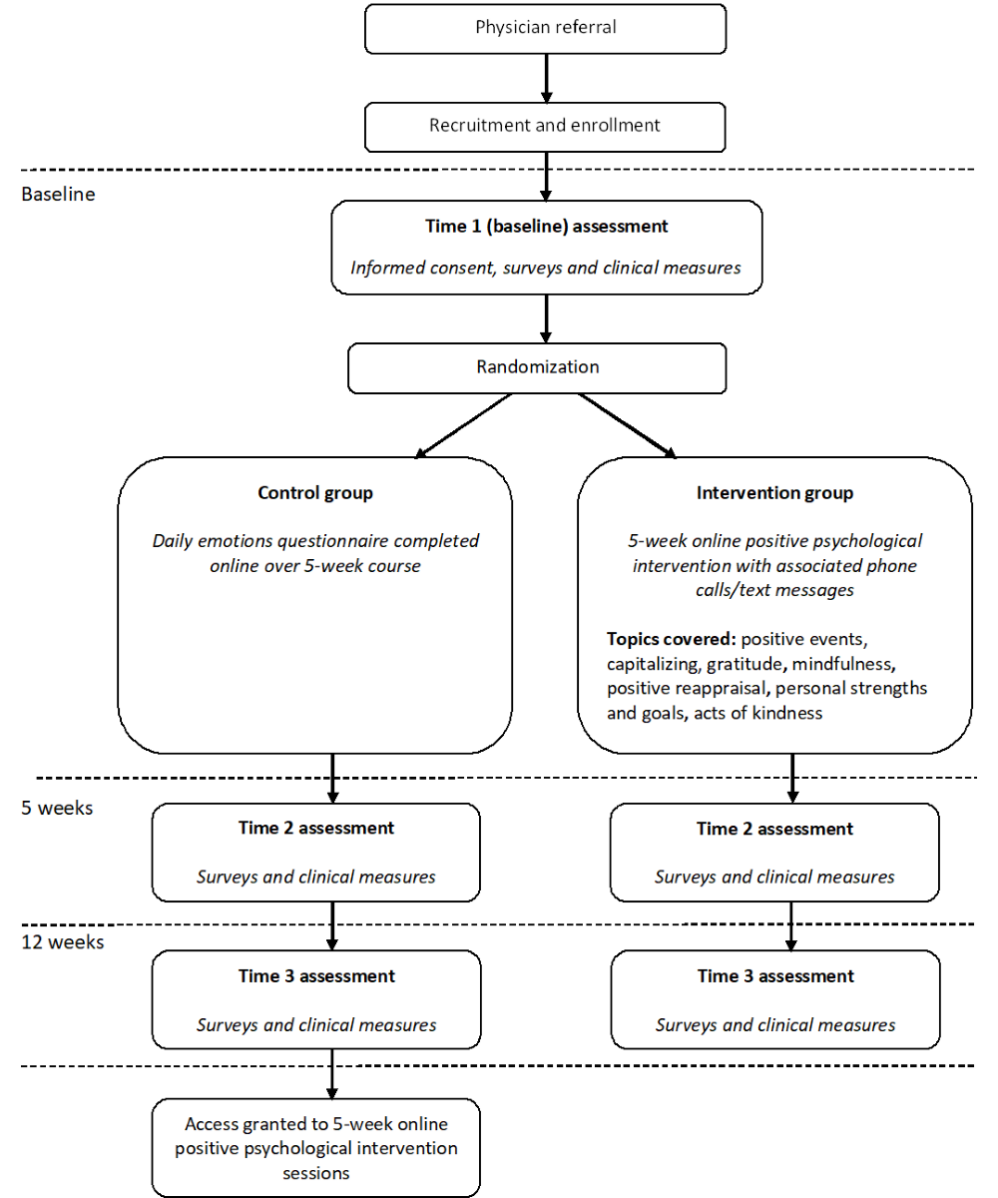
Recruitment and assessment timeline for the ¡Alégrate! web-based intervention.

Survey and clinical exams occur in tandem at 3 time points, as follows: Time 1 (baseline) at study enrollment; Time 2, immediately postintervention (5 weeks after baseline); and Time 3 (12 weeks after baseline). Figure 1 details the design of the study and the associated timeline for recruitment, enrollment, and assessment tasks.

Survey and clinical assessments are conducted in Spanish through in-person interviews by trained bilingual research staff in a one-to-one encounter at the participating FQHC. Questionnaires used and associated constructs assessed as part of the survey interview includes measures of psychological well-being (eg, optimism, positive affect), acculturation, self-reported health and medical comorbidities, engagement in healthy behaviors (eg, sodium intake, sleep quality, and duration), and medication adherence. Many survey instruments used in the current trial have undergone previous psychometric testing, showing adequate validity and reliability in the Spanish language. For those not previously translated, our team conducted thorough forward and backward translation procedures to craft a Spanish-language adaptation with linguistic equivalency. All survey data were collected using REDCap, a secure internet-based platform for building and managing web-based surveys. Finally, clinical examinations will be carried out by research staff to collect anthropometric data (eg, body mass index, waist-to-hip ratio), serum blood spots, sitting BP, and 24-hour ambulatory BP profiles. It should be noted that all data will be deidentified, stored in a password-protected computer, and/or stored in a locked cabinet in the principal investigator’s office.

After determining full eligibility, participants will be enrolled in the trial by providing written informed consent. After completing baseline assessments, participants will be randomly assigned in a 1:1 ratio to the intervention or active control condition using block randomization alternating between varying block sizes of 4, 6, or 8. The principal investigator created a random allocation table using Sealed Envelope, after which it was uploaded to REDCap to define the randomization model of *¡Alégrate!* to ensure proper blocking and stratification by sex (male and female) and use of antihypertensive medication (yes and no). A trained research staff generated allocation assignment through REDCap. It should be noted that REDCap conceals the allocation table and associated sequencing from research staff to prevent selection bias. Finally, given the pilot nature of a Phase II trial, blinding occurs only at the level of data analysis.

### Content of the Intervention

Our Spanish-language web-based *¡Alégrate!* positive psychological intervention was adapted from previously published and empirically validated curricula of the Moskowitz MARIGOLD trial [37-41]. Our 5-week *¡Alégrate!* intervention delivers curricular content via didactic instruction (eg, text, videos), journaling, and assigned at-home practice—all accessed via our website using investigator-purchased tablet computers. The website was designed and managed by Michael Cohn, a coinvestigator of the *¡Alégrate!* intervention. This 5-session, multicomponent web-based intervention instructs on 8 emotion regulation skills that have proven efficacious for increasing positive emotion and decreasing symptoms of depression and psychological distress in patients with varying chronic illness diagnoses, that is, those with metastatic breast cancer, HIV, and type 2 diabetes [38,40,41]. A detailed description of the curricula is published elsewhere [42]. Briefly, 8 empirically validated behavioral and cognitive skills are taught via our web-based interface, and include the following: (1) identification and use of personal strengths; (2) noticing of positive events in daily life; (3) prolonged appreciation and relishing of positive events; (4) positive reappraisal of stressful events or situations; (5) gratitude; (6) regular practice of mindfulness and meditation; (7) setting and working toward pragmatic and achievable goals; and (8) planning and performing acts of kindness. We previously tested the in-person delivery of MARIGOLD in the Spanish language [19].

**Figure 2:**
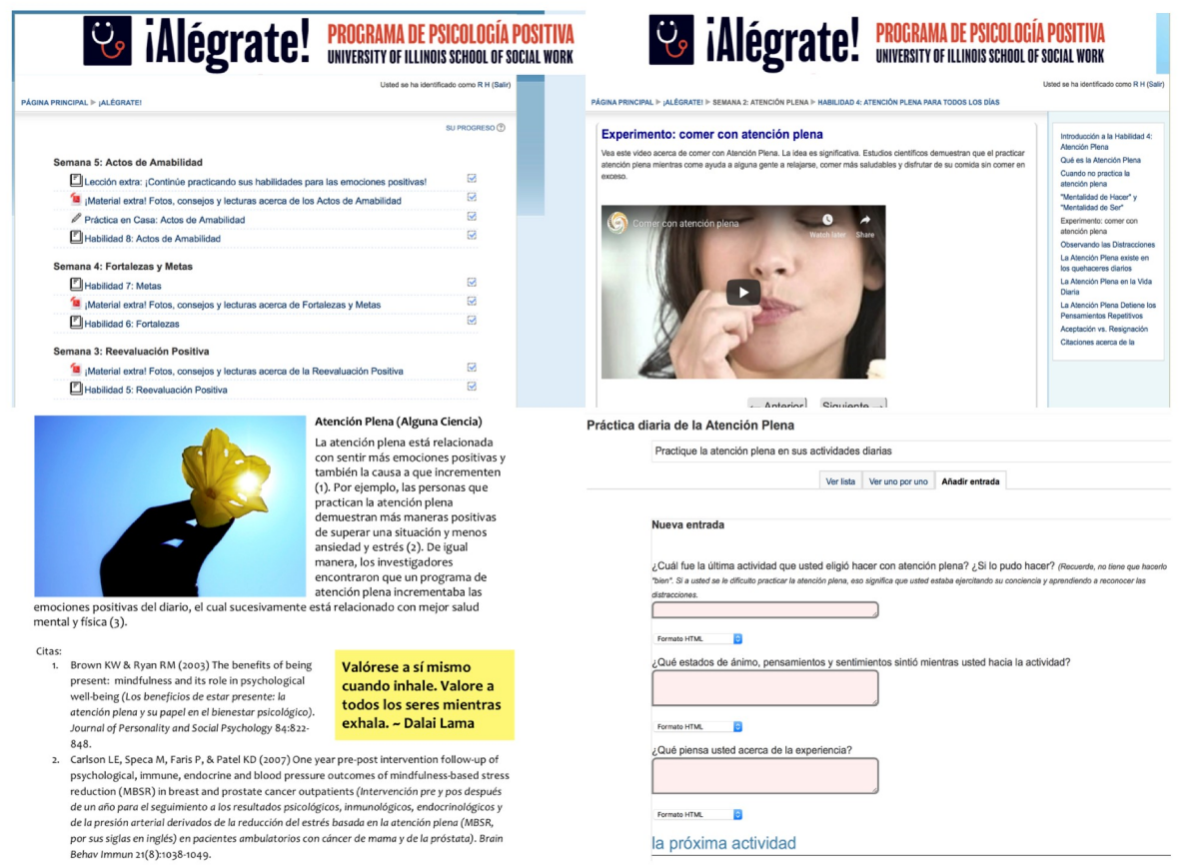
Screenshots of our Spanish-language internet-based positive psychological intervention accessed via a web browser.

During each weekly session, participants will learn skills and then complete the assigned at-home exercises to actively integrate the acquired techniques into daily life. As such, participants are instructed to log-in to the *¡Alégrate!* website for 30-min sessions at least three times per week. New skills are sequentially taught every week (see Table 1 for sequencing and content); to ensure successive instruction, new content becomes available only after a 7-day period, and once participants have completed the previous week’s content and at-home practice. Table 1 summarizes the content of the *¡Alégrate!* Spanish-language curricula, and Figure 2 presents screenshots of our web-based curricular interface [43]. Each participant logs in to the *¡Alégrate!* website using a unique username and password assigned by research staff—the tablet interface is customized with shortcuts for ease of navigation. Participants are also instructed to password protect their tablet to mitigate any data breaches from external parties.

In addition to accessing our web-based positive psychological intervention, participants will also receive weekly phone calls and text messages to reinforce lessons learned and as a strategy to maximize retention and continued visitation to our study site. Weekly phone calls will be brief, 10 to 15 min in duration, in which study staff review highlights of skills taught that week—along with technical support on any issues in handling the tablet computer or in navigating the *¡Alégrate!* site. Finally, participants with cell phone capability will receive a weekly text message from research staff highlighting the skills taught that week with the reminder to put them into daily practice—these sometimes take the form of a meme containing both images and text. For example, during Week 3 that focuses on mindfulness, they receive the following message:

Savoring requires that you slow down and pay attention to the positive events, large and small, that are happening in your day-to-day life. It can help you be more open, think more clearly, and appreciate things you have missed. It is important to be careful not to engage in thoughts that distract you from fully experiencing the present moment [La atención plena es una forma de prestar atención a los eventos positivos, grandes o pequeños, en su vida diaria. Lo puede ayudar a ser más abierto de mente, pensar más claramente y apreciar las cosas que quizás no noto. Es importante de no dedicarse en pensamientos que lo distraigan de experimentar plenamente el momento presente]

**Table 1:** Positive psychology skills imparted in the 5-week internet-based intervention.

Weekly session	Skills	Goals of the session
Week 1	Positive events and capitalizing (Skills 1 and 2)	Individuals are equipped to note positive life events in their day-to-day encounters. Capitalizing is an expressive response to positive events and includes telling others about it, marking the occurrence in some way, or even thinking about the even again later on
Week 1	Gratitude (Skill 3)	Gratitude is defined as a feeling of thankfulness and appreciation expressed toward others, which may include other people, nature, or God
Week 2	Mindfulness (Skill 4)	Mindfulness is defined as the ability to intentionally pay attention to and maintain nonjudgmental awareness of one’s thoughts, feelings, and physical sensations in the present moment
Week 3	Positive reappraisal (Skill 5)	Positive reappraisal is a form of coping in which the significance of the event is reinterpreted in a more positive way
Week 4	Focusing on personal strengths (Skill 6)	Identifying and focusing on one’s strengths as a form of self-affirmation to evaluate the resources possessed to cope with a stressful event
Week 4	Attainable goals (Skill 7)	Setting of realistic goals and imparting techniques to increase their progression and attainment
Week 5	Altruistic behaviors/acts of kindness (Skill 8)	Engagement in volunteerism and other altruistic behaviors

### Content of Control Sessions

Participants randomly assigned to the active control condition will be asked to visit the study site where they report their emotions (eg, grateful, happy, guilty, relieved, ashamed, etc), 3 times weekly, using the Differential Emotions Scale (DES), but they will not be granted access to curricular content of our *¡Alégrate!* positive psychological intervention. It takes 5 to 10 min to complete the DES online. Emotion reporting for the control condition occurs for the duration of the active intervention period, that is, 5 weeks in total. Research has previously established effectual use of emotion reporting as a control condition, with documented benefits similar to that of a placebo effect, and with high rates of retention (approximately 80%). Concerted efforts are undertaken to promote retention among participants in the control arm, including postcard notices and phone calls to remind participants to engage in web-based emotion reporting and to appear, in person, for scheduled clinical assessments. Finally, participants in the control condition will be granted access to the web-based *¡Alégrate!* curricular content upon completion of their final follow-up assessment at 12 weeks postbaseline.

Participants in both the treatment and control arms will receive detailed handouts on how to operate the *Samsung Galaxy Tab A* tablet computer, along with instructions on connecting to Wi-Fi and accessing the *¡Alégrate!* website. Handouts display textual instructions along with detailed step-by-step visual screenshots, along with a listing of Wi-Fi locations in the local neighborhood, for example, coffee shops and public libraries or bookstores. In addition, staff will hold, in person, 15- to 20-min tutorials at baseline to familiarize enrollees on tablet use, with the assurance of continual availability via phone or in person to assist with any issues and to troubleshoot difficulties encountered with the technology.

### Payment to the Participants

As compensation for their time invested and efforts in completing all trial-related tasks, participants in both trial arms (ie, treatment and active control) will be allowed to keep the investigator-purchased tablet computer at the end of the study period, which has a retail value of US $300. Participants who do not complete the full study, that is, those who drop out early, will be asked to return the tablet computer and will instead be given US $40 in cash for each assessment interview completed before dropping out. In addition, participants will receive reimbursement for travel-related costs (eg, parking, Uber, etc) and will be given light snacks while attending assessment interviews at the clinic site.

### Training and Intervention Fidelity

The principal investigator and all research staff are bilingual (English and Spanish) and bicultural (Mexican-American), and each underwent extensive training (minimum 30 hours) before recruitment and data collection. Research staff are trained to accurately implement the recruitment protocol, to properly deploy assessment interviews, and to support detailed tracking of participants throughout the 12-week study period. Over several weeks’ time, research staff were trained in the following key component areas: (1) knowledge in the protection and privacy of patient-related health information through UIUC’s Health Insurance Portability and Accountability Act Privacy and Security Compliance Program; (2) safe handling of Human Cell Lines/Materials in a Research Laboratory for the collection of blood spots, sponsored by the UIUC Institutional Biosafety Committee; (3) technological training, including electronic collection of survey data through REDCap, handling of tablet computers, and tracking of website visits and module completion across enrollees; (4) training in interview techniques, including building of rapport, interacting warmly and respectfully when working with Hispanic/Latino adults, that is, cultural value of *respeto*, consistency in ordering and wording of survey instruments, that is, quantitative methodology, (5) triggering of referrals to attending physician or distribution of resource listings when appropriate (eg, dangerously high levels of BP); and (6) protocol for weekly phone calls and text messages to reinforce web-based curricular content.

Multiple aspects of the intervention are manualized to strengthen the fidelity of the intervention and to ensure that the research staff are conveying identical content, particularly during weekly phone calls and when delivering messages via text. As such, research staff followed predrafted scripts, developed by the principal investigator, all of which followed a standard template as follows: (1) greet participants; (2) provide a brief summary (2-3 sentences) of the curricular content for that week; (3) from the participants’ perspective, inquire of important lessons learned from weekly content; (4) ask of any difficulties in using a tablet computer; and (5) remind participants that the staff are available via phone or email to troubleshoot if any questions or difficulties arise. All weekly text messages were prewritten in Spanish and sent to participants without modification.

Finally, research staff are trained on handling circumstances requiring immediate action and a patient referral to the attending physician. Research staff have detailed instructions, depicted using a graphic decision support tool (ie, decision tree), on the protocol to alert the attending physician if participants displayed elevated symptoms of depression (Center for Epidemiologic Studies Depression Scale ≥16; denoting probable clinical depression) or if they present with exorbitantly high BP levels, that is, ≥180/120 mm Hg. Training of research staff occurs at the outset during study startup, with booster sessions throughout the trial to ensure proficiency, along with immediate guidance when procedural issues are evident.

### Measures

#### Primary Outcome

Measures of sitting and ambulatory BP serve as the primary outcomes for the current *¡Alégrate!* trial. An ambulatory BP monitoring (ABPM) method will be used to capture 24-hour daytime and nighttime BP readings in the natural environment (Ultralite 90217A ABPM from Spacelabs Healthcare); this device has shown adequate reliability and accuracy. We will consider 24-hour mean systolic and diastolic readings. Weighing in at 9 ounces, the ABPM monitors were fitted and preprogramed to automatically inflate over a 24-hour period—specifically, measurements are taken every 30 min during daytime hours (7:00 AM to 11:00 PM) and every 60 min at night (11:00 PM to 6:00 AM). We will additionally use an automatic sphygmomanometer to evaluate sitting BP. This measurement device has been validated across multiple cohort studies, including MESA, NHANES, and HCHS/SOL. Three systolic and diastolic BP readings will be taken with participants in the seated position; mean values will be obtained by averaging the last 2 readings.

#### Secondary Outcomes

*Table 2* identifies the secondary outcomes and describes in detail the survey and clinical measures used to capture these constructs. Specifically, secondary outcomes include the following: (1) psychological well-being (depressive symptoms, perceived stress, positive and negative affect, optimism, emotional vitality, life engagement and meaning, happiness-inducing behavior, and social support), (2) healthy behaviors (physical activity, diet, sodium intake, smoking status, sleep quality and duration, and mediation use), and (3) biological materials (serum blood spots).

#### Antecedent Variables and Qualitative Data

In addition to the main outcomes of interest, we will also collect variables hypothesized to be antecedents or important confounders when exploring intervention effects. The list of antecedents or covariates is summarized in Table 2 and includes the following: (1) demographic factors, (2) anthropometric measurements, (3) acculturation, (4) level of religiosity, (5) self-reported mental and physical health, and (6) current or previous history of medical comorbidities. In addition, we will conduct qualitative exit interviews to capture facilitators and barriers in using the tablet computer to access *¡Alégrate!* curricular content. Indeed, qualitative process evaluation techniques further inform metrics of acceptability and utility for the modality of content distribution. Finally, we will also analyze data describing the overall use of the website by trial participants, including the number of website logins per participant, average length of screen time per site visit, and extent to which enrollees completed and recorded practicing of at-home exercises.

**Table 2:** Outcome, mediator, and intervention-based measures.

Measure					Description
**Outcomes (exams 1 through 3 unless otherwise noted)**					
	**Primary outcomes**				
		**Blood pressure measurements**			
			Sitting blood pressure	We will use an automatic sphygmomanometer to evaluate sitting BP. This measurement device has been validated across multiple cohort studies including MESA, NHANES, and HCHS/SOL [44,45]. The 3 systolic and diastolic blood pressure readings will be taken with participants in the seated position; mean values will be obtained by averaging the last 2 readings	
			24-hour ambulatory blood pressure	An ambulatory BP monitoring (ABPM) method will be used to capture 24-hour daytime and nighttime BP readings in the natural environment (CONTECTM Automatic Blood Pressure Monitor [ABPM50]); this device has shown adequate reliability and accuracy [46]. We will consider 24-hour mean systolic and diastolic readings. Weighing 1.87 lbs, the ABPM monitors will be fitted and pretested before 24-hour use [47]	
	**Secondary outcomes**				
		**Psychological well-being**			
			Depressive symptoms	The 20-item Center for Epidemiologic Studies Depression Scale (CES-D) will be used to measure depressive symptomatology [48]. The CES-D uses a 4-point Likert scale to probe the extent to which an individual has been troubled by depressive symptoms in the last 7 days; scores range from 0 to 60	
			Perceived stress scale (PSS)	The PSS includes 10-items to assess self-perceived levels of stress over the previous month using a Likert scale ranging from never to always [49]. Previously validated in the HCHS/SOL cohort [50], overall scores range from 0 to 40 for the full scale and includes items such as, “How often have you felt confident about your ability to handle your personal problems?”	
			Positive and negative affect	Participants will be asked to recall emotions experienced in the past week using a modified version of the Differential Emotions Scale [51]. A list of 26 different emotions will be provided (eg, grateful, happy, guilty, relieved, ashamed, or humiliated) and participants will be asked to identify how often they have experienced each on a scale ranging from 1—Not at all to 9—All the time	
			Dispositional optimism	The revised Life Orientation Test (*LOT-R*) will be used to assess dispositional optimism. The LOT-R is a validated 6-item self-administered questionnaire with possible scores ranging from 0 (least optimistic) to 24 (most optimistic) [52,53]. The scale includes 3 positively worded items and 3 negatively worded items that are rated on a 5-point Likert scale	
			Emotional vitality	Emotional vitality is characterized as a sense of overall well-being through active engagement in day-to-day activities and effectual regulation of emotions. Borrowing items from the General Well-being Schedule, this construct will be captured using a 6-item measure previously used in published studies with available evidence of adequate psychometric properties [54,55]. Respondents are asked to think about the previous 30 days, and using an ordinal scale are instructed to rate statements such as, “Has your daily life been full of things that were interesting to you?” and “Have you been feeling emotionally stable and sure of yourself?”	
			Life engagement and meaning (LET)	The LET is a 6-item instrument that probes the extent to which an individual engages in activities which they personally value and find meaningful [56], for example, *To me, the things that I do are all worthwhile.* Scores range from 6 to 30 with respondents rating items on a 5-point scale. Higher scores characterize an individual that experiences greater life engagement and purpose	
			Happiness-inducing behavior	This 38-item survey inquires of the extent to which participant engage in behavior or prescribed strategies known to induce happiness, for example, relaying gratitude, engaging in mediation and religious practices, focusing on positive life events, among others [57]	
			Self-perceived social support	The Medical Outcomes Study Social Support Survey will be used to quantify social support as provided by family, friends, and acquaintances [58]. This 20-item instrument first directs participants to quantify the total number of close friends and relatives they possess, that is, defined as the people they feel at ease with and can talk about what is on their mind. Remaining items ask participants to rate statements using a 5-point Likert scale that inquire of the perceived availability of support from family, friends, or others, if or when needed. Sample statements include “someone you can count on to listen to you when you need to talk” and “someone to help with daily chores if you were sick”	
		**Healthy behaviors**			
			Physical activity and dietary intake	Items of the Summary of Diabetes Self-Care Activities Measure will be used to assess engagement in physical activity and self-reported dietary intake. Items ask participants to report engagement across activities over a 7-day period and include queries such as, “On how many of the last SEVEN DAYS did you participate in at least 30 minutes of physical activity?” and “How many of the last SEVEN DAYS have you followed a healthful eating plan?”	
			Sodium intake	The Scored Sodium Questionnaire will be used to measure dietary intake specifically targeting quantification of sodium consumption [59]. Participants will be asked to report dietary patterns over the last 7 days across multiple food groups (eg, breads, processed meats, tinned or packet soups) with response options indicating daily consumption to rare or never eaten items. Dietary intake will also include items of the Summary of Diabetes Self-Care Activities questionnaire [60]	
			Smoking status	Participants will identify whether they are current smokers, former smokers, or if they have never smoked before. Current smokers will report the average number of cigarettes they smoke per day	
			Self-reported sleep quality and duration	Two self-report items were used to capture subjective rating of sleep. The first inquired about the number of hours per night of sleep (ie, during the main sleep period) that participants were getting on weekdays or workdays. The second asked participants to rate their typical night’s sleep during the past 4 weeks, with a Likert response option ranging from *very sound or restful* to *very restless*	
			Medication use and adherence	Participants will be asked if they were prescribed oral medication for their high blood pressure. Those indicating use of antihypertensive medication will be asked to identify the start date when they first initiated said use. We will also document whether participants had taken their blood pressure medication on the date of their clinical visit, and if so, the approximate time of day. Finally, medication adherence specific to antihypertensive drugs will be assessed using the Morisky Medication Adherence Scale [61]. At each scheduled assessment, participants will be instructed to bring all medications currently taken. Research staff will document details across medication (eg, dosage) and they will save a digital picture of associated pill bottles	
		**Markers of cardiovascular disease**			
			Serum blood spots	Serum blood spots will be collected from each participant at baseline and immediately postintervention (8 weeks). A trained research staff will prick the participant’s middle or ring finger using sterile procedures. After wiping away the first spot of blood, 5 subsequent drops will be collected using Whatman #903 filter paper. The blood spots will be stored for future analysis in a −20° freezer at the Institute for Minority Health Research at the University of Illinois at Chicago	
	**Covariates**				
		**Antecedent or confounding variables**			
			Demographic factors	We will gather basic demographic information from all participants including: age, sex, income, educational attainment, marital status, health insurance status, employment type, nativity status and number of years in the United States, and country of origin	
			Anthropometric measurements	Research staff will ascertain measures of height (to the nearest centimeter) and weight (to the nearest 0.1 kg) and will calculate BMI using these values. The waist-to-hip ratio will be derived from abdominal and hip girth obtained using a Gulick II 150- and 250-cm anthropometric tape measure with participants wearing light clothing	
			Acculturation/cultural factors	The Short Acculturation Scale for Hispanics will be used to capture the construct of acculturation in Hispanic/Latino adults [62]. The 10-item scale inquires of language use (Spanish vs English) across settings (home vs social life), language in which entertainment is consumed, and race/ethnicity of individuals across varied social circles. Derived from the Duke University Religion Index [63], religiosity will be assessed using a 5-items scale that includes questions such as, “My religious beliefs are what really lie behind my whole approach to life.” It also assesses frequency of attendance to church services and engagement in religious practices	
			Current health status and medical comorbidities	Self-reported physical and mental health will be measured using the 12-item Short Form Health Survey [64]. Participants will also self-report previous or current existence of any of the following medical conditions: heart attack; congestive heart failure; stroke; diabetes; arthritis; moderate or severe renal disease; fracture of the hip, wrist, arm, or shin; asthma; cirrhosis of the liver or liver disease; cancer; bypass of arteries in the leg; Parkinson’s disease; Alzheimer’s disease or dementia; HIV or AIDS; depression; or anxiety disorder	
			Overall website use	We will also analyze data describing overall use of the website by trial participants, including the number of website logins per participant, average length of screen time per site visit, and extent to which enrollees completed and recorded practicing of at-home exercises	

### Planned Analyses

The primary outcome of the *¡Alégrate!* trial focused on prospective changes in B*P* values, that is, both sitting BP and 24-hour ambulatory readings. The first aim was to examine the efficacy of the intervention in reducing B*P* values immediately postintervention (ie, 5 weeks) and at the 12-week follow-up period, that is, Exams 2 and 3. The second aim was to explore whether the intervention leads to significant improvements in psychological well-being and greater adherence to healthy behavioral practices. We will additionally test whether psychological well-being and healthy behaviors serve as mediators when exploring intervention effects on BP control. The third aim is to test intervention effects on circulating markers of inflammation, specifically high-sensitivity C-reactive protein.

Descriptive statistics, at baseline, will summarize participant characteristics for the full sample and stratified by intervention arm. Bivariate tests will be conducted to compare the intervention and control conditions to ensure compatibility across key variables (eg, demographic factors, well-being profiles, etc) and to test the success of the randomization protocol. Discrepancies in baseline values across conditions will inform covariates for inclusion when testing our main hypotheses of interest. In addition, we will document the number of missing observations over time to assess the potential for bias resulting from significant dropout and differential attrition by condition. We will perform independent sample two-tailed *t* tests and/or Fisher exact tests to compare baseline characteristics of enrollees who completed the trial versus those who prematurely dropped out, allowing us to test possible predictors of attrition.

Given our design and main objective of treatment evaluation, an intent-to-treat analysis will be conducted, where we will consider the data of all participants randomized, with retention of their original intervention assignment. Sensitivity analysis will use multiple imputation procedures across missing values to ensure inclusion of all observations, particularly those resulting from participants who withdraw, are lost to follow-up, or do not complete all assessments. In a supplementary analysis, we will use ordinary or logistic regression (as appropriate, given variable distribution) to implement a dose-response analysis to test whether participation-related factors (eg, number of sessions viewed, rate of homework completion) impact outcomes of interest.

We estimated the sample size requirements of a two-arm randomized trial to evaluate whether our web-based positive psychological intervention resulted in differential and clinically meaningful improvements in systolic BP of 6 mm Hg compared with an attention control condition. A repeated-measures design with systolic BP determination at baseline and 5 and 12 weeks was used. In addition, the following operating characteristics were assumed: (1) a between-subject standard deviation for BP of *σ=12 mm Hg*; (2) a within-subject correlation, *ρ=0.60* (3) a two-sided type I error probability of 0.05; and (4) 80% statistical power. Frison and Pocock [65] argue that adjusting for baseline measures (analysis of covariance) results in a more efficient analysis for a given sample size (larger statistical power). This approach yields an estimate of the required sample size of 28 participants per study arm under equal allocation. Furthermore, assuming a conservative attrition rate of 20%, an updated estimation produces the following sample size of participants per study arm.

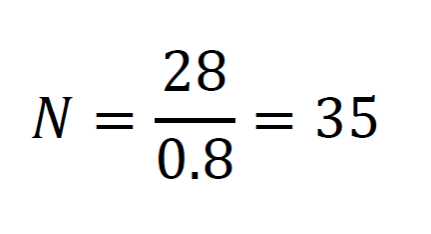

Stata 15 was used for sample size calculation (*sampsi* command) and all statistical analyses, with effect size estimation derived from published data on the clinical effectiveness of psychotherapeutic interventions to treat hypertension [47,66,67].

#### Aim 1: Determine the Efficacy of the ¡Alégrate! Web-Based Positive Psychological Intervention in Improving BP Control in Hispanic/Latino People With Hypertension From Baseline, Immediately Postintervention (5 Weeks), and at 12 Weeks After Baseline 

Independent samples *t* tests will be performed using 5-week systolic BP and diastolic BP as the dependent variables, with treatment condition serving as the grouping variable. Other model-based approaches to handle missing data, such as weighted estimating equations, will be implemented. We will also use mixed-effects models to compare changes in BP between the control and intervention arms as measured at baseline, 5 weeks, and 12 weeks. The independent variables include a time variable t (t=0, 5, 12), a dummy variable N (N=1, if PP intervention group; N=0, if attention control), and a cross-product term (t×N). For ambulatory data collected across a 24-hour period, sensitivity analyses will compare mean overall daytime versus nighttime values of BP. All data analyses were conducted using SAS 9.4 software (SAS Institute).

#### Aim 2: To Test Intervention Effects on Psychological Well-Being and Adherence to Healthy Behaviors and to Subsequently Explore Whether Improvements in Psychological Well-Being and Adherence to Healthy Behaviors Are Responsible for Better BP Control, That Is, Mediation Testing

Similar analytic techniques will be implemented for Aim 2 as used for Aim 1. The interaction of Group×Time will test if greater improvements are evident for psychological well-being and engagement in healthy behaviors (eg, dietary intake and self-reported physical activity) at 5 weeks postbaseline for the treatment arm as compared with the active control group. RM-ANOVA will also be used to examine whether the *¡Alégrate!* intervention is associated with greater improvements in psychological well-being and engagement in healthy behavior. In addition to reporting nominal *P* values, we will document the number of tests conducted and associated Bonferroni correction. Finally, we will conduct mediation analysis to test whether psychological well-being and healthy behaviors serve as intermediates (or mediators) through which *¡Alégrate!* impacts BP control. Tests of mediation will follow the recommendations of Shrout and Bolder to bootstrap product terms using Mplus. Given the dependency of indirect effects on the time interval, for multiwave models, we will compute overall, rather than time-specific, indirect effects.

#### Aim 3: Evaluate Intervention Effects on High-Sensitivity C-Reactive Protein

Similar analytic techniques as previously described will be used when testing intervention effects on chronic inflammation. Specifically, the term capturing Group×Time interaction will test whether lower levels of inflammation are evident at 8 weeks postbaseline for the intervention group versus the active control arm.

## Results

Activities of the Spanish-language *¡Alégrate!* intervention were funded in 2017, and data collection is ongoing. We expect to submit the trial results to peer-reviewed publications in 2021, soon after recruitment has been concluded and statistical analysis is finalized.

## Discussion

### Principal Findings

This paper describes the design and protocol of the *¡Alégrate!* program, which is a web-based positive psychological intervention tailored for Hispanic/Latino people with hypertension—with the curricular content and evidence-based skills disseminated fully in the Spanish language. Novel interventions specifically geared toward Hispanic/Latino adults are greatly needed, as this racial/ethnic group constitutes the largest minority group in the United States and they experience continued and ever-growing health disparities and high burden of cardiovascular disease risk factors [12,18]. As such, our Spanish-language web-based *¡Alégrate!* program has the potential to positively impact cardiovascular health profiles of Hispanic/Latino adults, particularly when it comes to BP control. Moreover, it offers a more cost-effective alternative to face-to-face delivery of our intervention through dissemination using a web-based platform—accessed through the comfort of an individual’s home.

### Strengths and Limitations

Limitations are evident in the design and eventual deployment of the *¡Alégrate!* web-based trial. Selective enrollment has the potential to introduce bias as Hispanic/Latino people with limited technological proficiency may decline to participate in the trial, or if enrolled, may have difficulty in connecting to Wi-Fi or in navigating the study site. Future trials may want to explore more user-friendly modalities that require little to no coaching that may be more appealing to less technologically savvy community members. We are recruiting in a Chicago-based neighborhood with a large population of Hispanic/Latino adults, the majority of which are of Mexican ancestry. Thus, the findings of the trial may not generalize to the wider, more heterogeneous Hispanic/Latino population across the United States with differences by country of origin, nativity status, and level of acculturation. Our design includes an active control condition where participants are asked to report their emotions, thrice weekly, via our web-based platform. This may not adequately adjust for effects derived from social support and contact with research staff as imparted in the treatment group. Future trials may want to deploy a more active control condition where participants’ access to web-based content is unrelated to the outcome of interest, for example, money management skills. Finally, blinding occurs only at the level of data analysis, and both trial participants and assessors (research staff collecting outcome data) are aware of randomization assignment, which can introduce unintended bias.

Despite these limitations, the strengths of our design are evident. We are the first to design and deliver a Spanish-language positive psychological intervention to Hispanic/Latino adults using a web-based platform. Second, we are collaborating with a Chicago-based clinic located in a neighborhood with residents predominantly of Hispanic/Latino origin, that is, approximately 21,500 Hispanic/Latino people, primarily of Mexican-American descent. Our partnering clinic is also a federally qualified site servicing the most vulnerable, including those with limited income and those with no health insurance. Finally, we provided all enrollees with an investigator-purchased tablet computer and developed detailed Spanish-language user guides (with screenshots) to assist participants in accessing our website, with research staff available via phone for additional tech support.

### Conclusions

Hispanic/Latino people are a vital population for testing our *¡Alégrate!* web-based programs due to an overwhelming need for interventions to improve cardiovascular health and evidence that psychological well-being may be a particularly relevant target. This is especially true within the context of a cultural group that values building of positive emotional bonds and encourages *personalismo* (ie, emphasis on agreeableness, politeness, or courtesy)—and, who despite these positive cultural inclinations, constitutes a collective group that experiences widening cardiac-related health disparities. Our team previously tested our positive psychological curriculum as delivered face-to-face by a psychologist/social worker in a group setting and in the Spanish language [19,42]. We found significant improvement when examining pre-post intervention changes in emotional vitality, subjective happiness, and engagement in happiness-inducing behaviors (eg, meditation). However, rates of retention were particularly low and often involved the inability to attend on-site sessions because of shifts in work schedules, inclement weather, and/or travel outside of the country. Offering an alternative to access our intervention content via a website, from the comfort of home and in a self-paced manner, may prove to be a viable and more effective alternative for Hispanic/Latino adults. This new delivery modality may also prove to be more cost-effective when compared with in-person delivery by a highly trained clinician.

As the use of technology continues to expand, it is imperative that vulnerable populations and ethnic/racial minorities are taken into account during its inception and expansion. How might technology be deployed within a more user-friendly interface for those with limited exposure to technology or those with little formal educational training who experience literacy challenges? For instance, the website for the current *¡Alégrate!* trial was created using inclusive design principles and usability testing to create a simple, straightforward interface to foster high rates of retention and acceptability. The home screen for our tablets was clutter-free with only 1 to 2 apps available that directly linked participants to our study website—with parameters imposed that restricted the addition of new apps and/or the visitation to entertainment sites, for example, Netflix and YouTube. Finally, websites and clinical apps developed from evidenced-based curricular content should be made available in the Spanish language, and other languages for mass and equitable consumption. Tech companies and their employees (eg, software engineers) will need to be creative to ensure that emerging technology is accessible to all—even those with limited technology experience, low literacy, and limited economic resources.

## Appendix

Multimedia Appendix 1 CONSORT-eHEALTH (V 1.6.1).

## Abbreviations

ABPM: ambulatory blood pressure monitoring
BP: blood pressure
CVD: cardiovascular disease
DES: Differential Emotions Scale
FQHC: Federally Qualified Health Center
NIH: National Institutes of Health
RCT: randomized controlled trial
UIUC: University of Illinois at Urbana-Champaign
