# Supplementary material for: A Web-Based Positive Psychological Intervention to Improve Blood Pressure Control in Spanish-Speaking Hispanic/Latino Adults With Uncontrolled Hypertension: Protocol and Design for the ¡Alégrate! Randomized Controlled Trial
Source: JMIR Res Protoc. 2020 Aug 4;9(8):e17721. doi: 10.2196/17721 (PMC7435608; doi:10.2196/17721)
Supplement: Multimedia Appendix 1 [file resprot_v9i8e17721_app1.pdf]

CONSORT-EHEALTH (V 1.6.1) - Submission/Publication Form

The CONSORT-EHEALTH checklist is intended for authors of randomized trials evaluating web-based and Internet-based applications/interventions, including mobile interventions, electronic games (incl multiplayer games), social media, certain telehealth applications, and other interactive and/or networked electronic applications. Some of the items (e.g. all subitems under item 5 - description of the intervention) may also be applicable for other study designs.

The goal of the CONSORT EHEALTH checklist and guideline is to be
a) a guide for reporting for authors of RCTs,
b) to form a basis for appraisal of an ehealth trial (in terms of validity)

CONSORT-EHEALTH items/subitems are MANDATORY reporting items for studies published in the Journal of Medical Internet Research and other journals / scientific societies endorsing the checklist.

Items numbered 1., 2., 3., 4a., 4b etc are original CONSORT or CONSORT-NPT (non-pharmacologic treatment) items.
Items with Roman numerals (i., ii, iii, iv etc.) are CONSORT-EHEALTH extensions/clarifications.

As the CONSORT-EHEALTH checklist is still considered in a formative stage, we would ask that you also RATE ON A SCALE OF 1-5 how important/useful you feel each item is FOR THE PURPOSE OF THE CHECKLIST and reporting guideline (optional).

Mandatory reporting items are marked with a red *.
In the textboxes, either copy & paste the relevant sections from your manuscript into this form - please include any quotes from your manuscript in QUOTATION MARKS,
or answer directly by providing additional information not in the manuscript, or elaborating on why the item was not relevant for this study.

YOUR ANSWERS WILL BE PUBLISHED AS A SUPPLEMENTARY FILE TO YOUR PUBLICATION IN JMIR AND ARE CONSIDERED PART OF YOUR PUBLICATION (IF ACCEPTED).
Please fill in these questions diligently. Information will not be copyedited, so please use proper spelling and grammar, use correct capitalization, and avoid abbreviations.

DO NOT FORGET TO SAVE AS PDF _AND_ CLICK THE SUBMIT BUTTON SO YOUR ANSWERS ARE IN OUR DATABASE !!!

Citation Suggestion (if you append the pdf as Appendix we suggest to cite this paper in the caption):

Eysenbach G, CONSORT-EHEALTH Group
CONSORT-EHEALTH: Improving and Standardizing Evaluation Reports of Web-based and Mobile Health Interventions
J Med Internet Res 2011;13(4):e126
URL: http://www.jmir.org/2011/4/e126/
doi: 10.2196/jmir.1923
PMID: 22209829

* Required

Your name *

First Last

Rosalba Hernandez

Primary Affiliation (short), City, Country *

University of Toronto, Toronto, Canada

University of Illinois, Urbana-Champaign, USA

Your e-mail address *

abc@gmail.com

rherna17@illinois.edu

Title of your manuscript *

Provide the (draft) title of your manuscript.

Web-based Positive Psychological Intervention to Improve Blood Pressure Control in Spanish-speaking Hispanic/Latino Adults with Uncontrolled Hypertension: Protocol and Design of the ¡Alégrate! Randomized Controlled Trial

Name of your App/Software/Intervention *

If there is a short and a long/alternate name, write the short name first and add the long name in brackets.

¡Alégrate!

Evaluated Version (if any)

e.g. "V1", "Release 2017-03-01", "Version 2.0.27913"

V2 Released 2020-04-17

Language(s) *

What language is the intervention/app in? If multiple languages are available, separate by comma (e.g. "English, French")

Spanish

URL of your Intervention Website or App

e.g. a direct link to the mobile app on app in appstore (itunes, Google Play), or URL of the website. If the intervention is a DVD or hardware, you can also link to an Amazon page.

https://www.marigoldstudy.org/alegrate/login,

URL of an image/screenshot (optional)

Your answer

Accessibility *

Can an enduser access the intervention presently?

- ○ access is free and open
- ◉ access only for special usergroups, not open
- ○ access is open to everyone, but requires payment/subscription/in-app purchases
- ○ app/intervention no longer accessible
- ○ Other:

Primary Medical Indication/Disease/Condition *

e.g. "Stress", "Diabetes", or define the target group in brackets after the condition, e.g. "Autism (Parents of children with)", "Alzheimers (Informal Caregivers of)"

Hypertension

Primary Outcomes measured in trial *

comma-separated list of primary outcomes reported in the trial

"Measures of sitting and ambulatory blood pre

Secondary/other outcomes

Are there any other outcomes the intervention is expected to affect?

"Specifically, secondary outcomes include: a) psychological well-being [depressive symptoms, perceived stress, positive and negative affect, optimism, emotional vitality, life engagement and meaning, happiness inducing behavior, and social support], (2) health behaviors [physical activity, diet, sodium intake, smoking status, sleep quality and duration, and mediation use], and (3) biological materials [serum blood spots]."

Recommended "Dose" *

What do the instructions for users say on how often the app should be used?

- [x] Approximately Daily
- [ ] Approximately Weekly
- [ ] Approximately Monthly
- [ ] Approximately Yearly
- [ ] "as needed"
- [ ] Other:

Approx. Percentage of Users (starters) still using the app as recommended after 3 months *

- (selected) unknown / not evaluated
- 0-10%
- 11-20%
- 21-30%
- 31-40%
- 41-50%
- 51-60%
- 61-70%
- 71%-80%
- 81-90%
- 91-100%
- Other:

Overall, was the app/intervention effective? *

- ( ) yes: all primary outcomes were significantly better in intervention group vs control
- ( ) partly: SOME primary outcomes were significantly better in intervention group vs control
- ( ) no statistically significant difference between control and intervention
- ( ) potentially harmful: control was significantly better than intervention in one or more outcomes
- ( ) inconclusive: more research is needed
- (x) Other: Data collection is on-going.

Article Preparation Status/Stage *

At which stage in your article preparation are you currently (at the time you fill in this form)

- ( ) not submitted yet - in early draft status
- ( ) not submitted yet - in late draft status, just before submission
- ( ) submitted to a journal but not reviewed yet
- (x) submitted to a journal and after receiving initial reviewer comments
- ( ) submitted to a journal and accepted, but not published yet
- ( ) published
- ( ) Other:

Journal *

If you already know where you will submit this paper (or if it is already submitted), please provide the journal name (if it is not JMIR, provide the journal name under "other")

- ○ not submitted yet / unclear where I will submit this
- ◉ Journal of Medical Internet Research (JMIR)
- ○ JMIR mHealth and UHealth
- ○ JMIR Serious Games
- ○ JMIR Mental Health
- ○ JMIR Public Health
- ○ JMIR Formative Research
- ○ Other JMIR sister journal
- ○ Other:

Is this a full powered effectiveness trial or a pilot/feasibility trial? *

- ◉ Pilot/feasibility
- ○ Fully powered

Manuscript tracking number *

If this is a JMIR submission, please provide the manuscript tracking number under "other" (The ms tracking number can be found in the submission acknowledgement email, or when you login as author in JMIR. If the paper is already published in JMIR, then the ms tracking number is the four-digit number at the end of the DOI, to be found at the bottom of each published article in JMIR)

- ○ no ms number (yet) / not (yet) submitted to / published in JMIR
- ◉ Other: ms #17721

TITLE AND ABSTRACT

1a) TITLE: Identification as a randomized trial in the title

1a) Does your paper address CONSORT item 1a? *

I.e does the title contain the phrase "Randomized Controlled Trial"? (if not, explain the reason under "other")

- (x) yes
- ( ) Other:

1a-i) Identify the mode of delivery in the title

Identify the mode of delivery. Preferably use “web-based” and/or “mobile” and/or “electronic game” in the title. Avoid ambiguous terms like “online”, “virtual”, “interactive”. Use “Internet-based” only if Intervention includes non-web-based Internet components (e.g. email), use “computer-based” or “electronic” only if offline products are used. Use “virtual” only in the context of “virtual reality” (3-D worlds). Use “online” only in the context of “online support groups”. Complement or substitute product names with broader terms for the class of products (such as “mobile” or “smart phone” instead of “iphone”), especially if the application runs on different platforms.

	1	2	3	4	5	
subitem not at all important	( )	( )	( )	( )	(x)	essential

Does your paper address subitem 1a-i? *

Copy and paste relevant sections from manuscript title (include quotes in quotation marks "like this" to indicate direct quotes from your manuscript), or elaborate on this item by providing additional information not in the ms, or briefly explain why the item is not applicable/relevant for your study

"Web-based Positive Psychological Intervention"

1a-ii) Non-web-based components or important co-interventions in title

Mention non-web-based components or important co-interventions in title, if any (e.g., “with telephone support”).

	1	2	3	4	5	
subitem not at all important	○	○	○	○	○	essential

Does your paper address subitem 1a-ii?

Copy and paste relevant sections from manuscript title (include quotes in quotation marks "like this" to indicate direct quotes from your manuscript), or elaborate on this item by providing additional information not in the ms, or briefly explain why the item is not applicable/relevant for your study

Your answer

1a-iii) Primary condition or target group in the title

Mention primary condition or target group in the title, if any (e.g., “for children with Type I Diabetes”) Example: A Web-based and Mobile Intervention with Telephone Support for Children with Type I Diabetes: Randomized Controlled Trial

	1	2	3	4	5	
subitem not at all important	○	○	○	○	◉	essential

Does your paper address subitem 1a-iii? *

Copy and paste relevant sections from manuscript title (include quotes in quotation marks "like this" to indicate direct quotes from your manuscript), or elaborate on this item by providing additional information not in the ms, or briefly explain why the item is not applicable/relevant for your study

"Spanish-speaking Hispanic/Latino Adults with Uncontrolled Hypertension"

1b) ABSTRACT: Structured summary of trial design, methods, results, and conclusions

NPT extension: Description of experimental treatment, comparator, care providers, centers, and blinding status.

1b-i) Key features/functionalities/components of the intervention and comparator in the METHODS section of the ABSTRACT

Mention key features/functionalities/components of the intervention and comparator in the abstract. If possible, also mention theories and principles used for designing the site. Keep in mind the needs of systematic reviewers and indexers by including important synonyms. (Note: Only report in the abstract what the main paper is reporting. If this information is missing from the main body of text, consider adding it)

	1	2	3	4	5	
subitem not at all important	○	○	○	○	◉	essential

Does your paper address subitem 1b-i? *

Copy and paste relevant sections from the manuscript abstract (include quotes in quotation marks "like this" to indicate direct quotes from your manuscript), or elaborate on this item by providing additional information not in the ms, or briefly explain why the item is not applicable/relevant for your study

"Enrollees will be randomly assigned to one of two trial arms: (1) Web-based positive psychological intervention, or (2) an active control condition (e.g., thrice weekly emotion reporting). Our 5-week Spanish-language ¡Alégrate! intervention is Web-based and delivers curricular content via didactic instruction (e.g., text, videos), journaling, and assigned at-home practice—all accessed online using investigator-purchased tablet computers, with unique username and passwords assigned to each enrollee. Targeted skills of ¡Alégrate! include noting daily positive events, positive reappraisal of stressful events, effective expression of gratitude, performing acts of kindness, and regular practice of mindfulness and meditation, among others."

1b-ii) Level of human involvement in the METHODS section of the ABSTRACT

Clarify the level of human involvement in the abstract, e.g., use phrases like “fully automated” vs. “therapist/nurse/care provider/physician-assisted” (mention number and expertise of providers involved, if any). (Note: Only report in the abstract what the main paper is reporting. If this information is missing from the main body of text, consider adding it)

	1	2	3	4	5	
subitem not at all important	○	○	○	○	○	essential

Does your paper address subitem 1b-ii?

Copy and paste relevant sections from the manuscript abstract (include quotes in quotation marks "like this" to indicate direct quotes from your manuscript), or elaborate on this item by providing additional information not in the ms, or briefly explain why the item is not applicable/relevant for your study

"Our 5-week Spanish-language ¡Alégrate! intervention is Web-based and delivers curricular content via didactic instruction (e.g., text, videos), journaling, and assigned at-home practice—all accessed online using investigator-purchased tablet computers, with unique username and passwords assigned to each enrollee."

"The outcomes of interest are collected by trained research staff through in-person interviews using REDCap software."

1b-iii) Open vs. closed, web-based (self-assessment) vs. face-to-face assessments in the METHODS section of the ABSTRACT

Mention how participants were recruited (online vs. offline), e.g., from an open access website or from a clinic or a closed online user group (closed usergroup trial), and clarify if this was a purely web-based trial, or there were face-to-face components (as part of the intervention or for assessment). Clearly say if outcomes were self-assessed through questionnaires (as common in web-based trials). Note: In traditional offline trials, an open trial (open-label trial) is a type of clinical trial in which both the researchers and participants know which treatment is being administered. To avoid confusion, use “blinded” or “unblinded” to indicated the level of blinding instead of “open”, as “open” in web-based trials usually refers to “open access” (i.e. participants can self-enrol). (Note: Only report in the abstract what the main paper is reporting. If this information is missing from the main body of text, consider adding it)

	1	2	3	4	5	
subitem not at all important	○	○	○	○	○	essential

Does your paper address subitem 1b-iii?

Copy and paste relevant sections from the manuscript abstract (include quotes in quotation marks "like this" to indicate direct quotes from your manuscript), or elaborate on this item by providing additional information not in the ms, or briefly explain why the item is not applicable/relevant for your study

"The outcomes of interest are collected by trained research staff through in-person interviews using REDCap software."

1b-iv) RESULTS section in abstract must contain use data

Report number of participants enrolled/assessed in each group, the use/uptake of the intervention (e.g., attrition/adherence metrics, use over time, number of logins etc.), in addition to primary/secondary outcomes. (Note: Only report in the abstract what the main paper is reporting. If this information is missing from the main body of text, consider adding it)

	1	2	3	4	5	
subitem not at all important	○	○	○	○	○	essential

Does your paper address subitem 1b-iv?

Copy and paste relevant sections from the manuscript abstract (include quotes in quotation marks "like this" to indicate direct quotes from your manuscript), or elaborate on this item by providing additional information not in the ms, or briefly explain why the item is not applicable/relevant for your study

"Activities of the ¡Alégrate! intervention were funded in 2017, and data collection is on-going. We expect to submit trial results for peer-reviewed publication in 2021, soon after recruitment has concluded and statistical analyses are finalized."

1b-v) CONCLUSIONS/DISCUSSION in abstract for negative trials

Conclusions/Discussions in abstract for negative trials: Discuss the primary outcome - if the trial is negative (primary outcome not changed), and the intervention was not used, discuss whether negative results are attributable to lack of uptake and discuss reasons. (Note: Only report in the abstract what the main paper is reporting. If this information is missing from the main body of text, consider adding it)

	1	2	3	4	5	
subitem not at all important	○	○	○	○	○	essential

Does your paper address subitem 1b-v?

Copy and paste relevant sections from the manuscript abstract (include quotes in quotation marks "like this" to indicate direct quotes from your manuscript), or elaborate on this item by providing additional information not in the ms, or briefly explain why the item is not applicable/relevant for your study

Not applicable; data collection is on-going.

INTRODUCTION

2a) In INTRODUCTION: Scientific background and explanation of rationale

2a-i) Problem and the type of system/solution

Describe the problem and the type of system/solution that is object of the study: intended as stand-alone intervention vs. incorporated in broader health care program? Intended for a particular patient population? Goals of the intervention, e.g., being more cost-effective to other interventions, replace or complement other solutions? (Note: Details about the intervention are provided in “Methods” under 5)

	1	2	3	4	5	
subitem not at all important	○	○	○	○	◉	essential

Does your paper address subitem 2a-i? *

Copy and paste relevant sections from the manuscript (include quotes in quotation marks "like this" to indicate direct quotes from your manuscript), or elaborate on this item by providing additional information not in the ms, or briefly explain why the item is not applicable/relevant for your study

"Mounting evidence suggests that positive psychological well-being (hereafter, psychological well-being)—which includes positively valenced feelings or cognitive appraisals such as happiness, optimism, and purpose in life—is independently associated with favorable cardiac health, e.g., reduced risk for incident hypertension, better lipid profiles, reduced inflammatory markers, and reduced odds of incident heart disease and cardiac related mortality [5-7]. Nevertheless, while multiple studies link psychological well-being with better cardiac health [6-8] there is a need to investigate the most effective approaches to cultivate psychological well-being in an effort to reduce cardiac risk."

"Additional research is needed, however, to explore strategies that boost psychological well-being specifically in Hispanic/Latino adults and whether this leads to better heart health and blood pressure management. To-date, pilot trials highlight that Hispanic/Latino adults find interventions targeting psychological well-being both enjoyable and of benefit [19, 20]."

"About 22% [15] of Hispanics/Latinos have hypertension, and they tend to display lower compliance with treatment recommendations when compared to their non-Hispanic peers [15, 17]."

"There are no specific clinical CVD guidelines addressing the unique characteristics of Hispanics/Latinos; which highlights the need for novel and more effective disease management efforts for this racial/ethnic group [14]."

"Recent studies have used Web-based interventions to promote psychological well-being in different patient populations [26]. For example, Web-based delivery formats were found to be effective in the management of symptoms of depression, anxiety and stress [30-34] in healthy and clinical populations. However, most of these studies have been conducted in non-Hispanic white samples. There is a need for better representation of underserved, racial/ethnic minorities, in Web-based psychological well-being interventions."

"Empirical evidence suggests that effect sizes for Web-based therapies are similar to those deployed using traditional face-to-face approaches, with high patient satisfaction reported across these hi-tech platforms. Few evidenced-based programs, however, are developed specifically for and deployed with Hispanic/Latino adults using Web-based platforms."

"¡Alégrate! [Be Happy!], is a Phase II randomized controlled trial (RCT) created to address these limitations by testing initial efficacy of a Spanish-language Web-based intervention. The RCT intends to boost psychological well-being in Spanish-speaking Hispanic/Latino adults with hypertension by examining changes in BP, psychological well-being, health behavior adherence, and circulating serum inflammation."

2a-ii) Scientific background, rationale: What is known about the (type of) system

Scientific background, rationale: What is known about the (type of) system that is the object of the study (be sure to discuss the use of similar systems for other conditions/diagnoses, if appropiate), motivation for the study, i.e. what are the reasons for and what is the context for this specific study, from which stakeholder viewpoint is the study performed, potential impact of findings [2]. Briefly justify the choice of the comparator.

	1	2	3	4	5	
subitem not at all important	○	○	○	○	◉	essential

Does your paper address subitem 2a-ii? *

Copy and paste relevant sections from the manuscript (include quotes in quotation marks "like this" to indicate direct quotes from your manuscript), or elaborate on this item by providing additional information not in the ms, or briefly explain why the item is not applicable/relevant for your study

"Observational evidence links psychological well-being with better overall health, more healthful coping tendencies, improved quality of life, and healthy longevity [8, 21]. Psychological well-being focuses on positive thoughts and feelings at the individual-level, which may be key in promoting health behaviors in hypertension management [8]. Evidence suggests that psychological well-being is related to healthier blood pressure (BP) profiles and plays a protective role in disease incidence [22, 23]. In over 1,000 healthy non-Hispanic adults, a large prospective study found that higher levels of psychological well-being were associated with decreased likelihood of incident hypertension at a one-year follow-up [24]. In 126 British civil servants, aggregate baseline levels of happiness were negatively associated with year 3 measures of systolic blood pressure, independent of known confounders."

"The use of Web-based interventions in different clinical populations has grown significantly [26]. Web-based interventions tend to be less costly than face-to-face designs, offering flexibility, and greater access [27]. Internet use has also grown substantially among Hispanic/Latino adults, with 80% reporting use of the internet [27]. A majority of Hispanic/Latinos report having a computer (78%), and report owning a smartphone (71%) [28]. Web-based interventions have the potential for broad dissemination and may help eliminate common barriers to participation (e.g. transportation, scheduling conflict, and lack of child care) [29]. Recent studies have used Web-based interventions to promote psychological well-being in different patient populations [26]. For example, Web-based delivery formats were found to be effective in the management of symptoms of depression, anxiety and stress [30-34] in healthy and clinical populations. However, most of these studies have been conducted in non-Hispanic white samples. There is a need for better representation of underserved, racial/ethnic minorities, in Web-based psychological well-being interventions."

"Empirical evidence suggests that effect sizes for Web-based therapies are similar to those deployed using traditional face-to-face approaches, with high patient satisfaction reported across these hi-tech platforms. Few evidenced-based programs, however, are developed specifically for and deployed with Hispanic/Latino adults using Web-based platforms. Schueller et al. (2019) identified only six studies deploying digital health technologies in Hispanic/Latino populations [35]."

2b) In INTRODUCTION: Specific objectives or hypotheses

Does your paper address CONSORT subitem 2b? *

Copy and paste relevant sections from the manuscript (include quotes in quotation marks "like this" to indicate direct quotes from your manuscript), or elaborate on this item by providing additional information not in the ms, or briefly explain why the item is not applicable/relevant for your study

"The RCT intends to boost psychological well-being in Spanish-speaking Hispanic/Latino adults with hypertension by examining changes in BP, psychological well-being, health behavior adherence, and circulating serum inflammation. This Web-based intervention is built on activities that target psychological well-being by promoting optimism, gratitude, and positive affect directly though activities such as recalling positive life events, identifying and employing personal strengths, and engaging in acts of kindness, among others. We hypothesized that compared to participants in the control condition, those in the active intervention group will show greater improvements in BP, higher scores for psychological well-being, greater engagement in healthful behaviors, and lower levels of inflammatory makers at follow up."

METHODS

3a) Description of trial design (such as parallel, factorial) including allocation ratio

Does your paper address CONSORT subitem 3a? *

Copy and paste relevant sections from the manuscript (include quotes in quotation marks "like this" to indicate direct quotes from your manuscript), or elaborate on this item by providing additional information not in the ms, or briefly explain why the item is not applicable/relevant for your study

"As such, the current Phase II pilot trial implements a parallel group design with a 1:1 allocation ratio—with two fixed factors: a) sex [male, female] and b) antihypertensive medication use [yes, no]."

3b) Important changes to methods after trial commencement (such as eligibility criteria), with reasons

Does your paper address CONSORT subitem 3b? *

Copy and paste relevant sections from the manuscript (include quotes in quotation marks "like this" to indicate direct quotes from your manuscript), or elaborate on this item by providing additional information not in the ms, or briefly explain why the item is not applicable/relevant for your study

Not applicable; no issues have arrived to require change in eligibility criteria.

3b-i) Bug fixes, Downtimes, Content Changes

Bug fixes, Downtimes, Content Changes: ehealth systems are often dynamic systems. A description of changes to methods therefore also includes important changes made on the intervention or comparator during the trial (e.g., major bug fixes or changes in the functionality or content) (5-iii) and other “unexpected events” that may have influenced study design such as staff changes, system failures/downtimes, etc. [2].

	1	2	3	4	5	
subitem not at all important	○	○	○	○	○	essential

Does your paper address subitem 3b-i?

Copy and paste relevant sections from the manuscript (include quotes in quotation marks "like this" to indicate direct quotes from your manuscript), or elaborate on this item by providing additional information not in the ms, or briefly explain why the item is not applicable/relevant for your study

Not applicable; there has been no change to methods nor problems with the "¡Alégrate!" website that has required bug fixes, downtimes, or changes to content.

4a) Eligibility criteria for participants

Does your paper address CONSORT subitem 4a? *

Copy and paste relevant sections from the manuscript (include quotes in quotation marks "like this" to indicate direct quotes from your manuscript), or elaborate on this item by providing additional information not in the ms, or briefly explain why the item is not applicable/relevant for your study

"Spanish-speaking Hispanic/Latino adults are eligible to enroll in the ¡Alégrate! trial if they meet the following criteria: 1) Hispanic/Latino heritage based on self-report, 2) aged 18 years or older, 3) fluent in Spanish, i.e., ability to read, speak, and write Spanish, 4) elevated sitting BP (≥140/90), and 5) basic technological literacy with the ability to access internet at home or in a public setting."

4a-i) Computer / Internet literacy

Computer / Internet literacy is often an implicit “de facto” eligibility criterion - this should be explicitly clarified.

	1	2	3	4	5	
subitem not at all important	○	○	○	○	◉	essential

Does your paper address subitem 4a-i?

Copy and paste relevant sections from the manuscript (include quotes in quotation marks "like this" to indicate direct quotes from your manuscript), or elaborate on this item by providing additional information not in the ms, or briefly explain why the item is not applicable/relevant for your study

"5) basic technological literacy with the ability to access internet at home or in a public setting."

4a-ii) Open vs. closed, web-based vs. face-to-face assessments:

Open vs. closed, web-based vs. face-to-face assessments: Mention how participants were recruited (online vs. offline), e.g., from an open access website or from a clinic, and clarify if this was a purely web-based trial, or there were face-to-face components (as part of the intervention or for assessment), i.e., to what degree got the study team to know the participant. In online-only trials, clarify if participants were quasi-anonymous and whether having multiple identities was possible or whether technical or logistical measures (e.g., cookies, email confirmation, phone calls) were used to detect/prevent these.

	1	2	3	4	5	
subitem not at all important	○	○	○	○	◉	essential

Does your paper address subitem 4a-ii? *

Copy and paste relevant sections from the manuscript (include quotes in quotation marks "like this" to indicate direct quotes from your manuscript), or elaborate on this item by providing additional information not in the ms, or briefly explain why the item is not applicable/relevant for your study

"The attending physician and clinical staff (e.g., registered nurse, medical assistant) identify potentially eligible patients and subsequently refers them to ¡Alégrate! research staff who then follow-up via phone or in-person; participant information is stored in a secure on-line portal."

"Research staff will also approach patients in the waiting area to provide information about the research study and offer an invitation to receive a no-cost blood pressure screening; research staff will collect contact information of those expressing interest to determine full eligibility."

4a-iii) Information giving during recruitment

Information given during recruitment. Specify how participants were briefed for recruitment and in the informed consent procedures (e.g., publish the informed consent documentation as appendix, see also item X26), as this information may have an effect on user self-selection, user expectation and may also bias results.

	1	2	3	4	5	
subitem not at all important	○	○	○	○	○	essential

Does your paper address subitem 4a-iii?

Copy and paste relevant sections from the manuscript (include quotes in quotation marks "like this" to indicate direct quotes from your manuscript), or elaborate on this item by providing additional information not in the ms, or briefly explain why the item is not applicable/relevant for your study

"The consent form and study materials outline the design of the study and inform participants of random assignment into one of two groups, either the group where “they learn skills to boost their mood and positive emotions” or the group where “they would report their emotions on a thrice-weekly basis.” It is communicated that all curricular instruction occurs on-line by visiting our ¡Alégrate! website. Additionally, staff inform participants that three in-person clinical visits are required, each 2-3 hour in length, where staff collect survey and clinical data, including collection of anthropometric information, blood spots, and 24-hour blood pressure monitoring; home visits are also offered for data collection to increase rates of retention. Participants are informed that they keep the investigator-purchased tablet computer at the end of the study as compensation for their participation and time invested [37]."

4b) Settings and locations where the data were collected

Does your paper address CONSORT subitem 4b? *

Copy and paste relevant sections from the manuscript (include quotes in quotation marks "like this" to indicate direct quotes from your manuscript), or elaborate on this item by providing additional information not in the ms, or briefly explain why the item is not applicable/relevant for your study

"Additionally, staff inform participants that three in-person clinical visits are required, each 2-3 hour in length, where staff collect survey and clinical data, including collection of anthropometric information, blood spots, and 24-hour blood pressure monitoring; home visits are also offered for data collection to increase rates of retention."

4b-i) Report if outcomes were (self-)assessed through online questionnaires

Clearly report if outcomes were (self-)assessed through online questionnaires (as common in web-based trials) or otherwise.

	1	2	3	4	5	
subitem not at all important	○	○	○	○	◉	essential

Does your paper address subitem 4b-i? *

Copy and paste relevant sections from the manuscript (include quotes in quotation marks "like this" to indicate direct quotes from your manuscript), or elaborate on this item by providing additional information not in the ms, or briefly explain why the item is not applicable/relevant for your study

"Survey and clinical assessments are conducted in Spanish through in-person interviews by trained bilingual research staff in a one-to-one encounter at the participating FQHC."

4b-ii) Report how institutional affiliations are displayed

Report how institutional affiliations are displayed to potential participants [on ehealth media], as affiliations with prestigious hospitals or universities may affect volunteer rates, use, and reactions with regards to an intervention.(Not a required item – describe only if this may bias results)

	1	2	3	4	5	
subitem not at all important	○	○	○	○	○	essential

Does your paper address subitem 4b-ii?

Copy and paste relevant sections from the manuscript (include quotes in quotation marks "like this" to indicate direct quotes from your manuscript), or elaborate on this item by providing additional information not in the ms, or briefly explain why the item is not applicable/relevant for your study

Your answer

5) The interventions for each group with sufficient details to allow replication, including how and when they were actually administered

5-i) Mention names, credential, affiliations of the developers, sponsors, and owners

Mention names, credential, affiliations of the developers, sponsors, and owners [6] (if authors/evaluators are owners or developer of the software, this needs to be declared in a "Conflict of interest" section or mentioned elsewhere in the manuscript).

	1	2	3	4	5	
subitem not at all important	○	○	○	○	○	essential

Does your paper address subitem 5-i?

Copy and paste relevant sections from the manuscript (include quotes in quotation marks "like this" to indicate direct quotes from your manuscript), or elaborate on this item by providing additional information not in the ms, or briefly explain why the item is not applicable/relevant for your study

"Our Spanish-language Web-based ¡Alégrate! positive psychological intervention was adapted from previously published and empirically validated curricula of Moskowitz's MARIGOLD trial [38-42]."

"The website was designed and managed by Michael Cohn, a co-investigator of ¡Alégrate!."

5-ii) Describe the history/development process

Describe the history/development process of the application and previous formative evaluations (e.g., focus groups, usability testing), as these will have an impact on adoption/use rates and help with interpreting results.

	1	2	3	4	5	
subitem not at all important	○	○	○	○	○	essential

Does your paper address subitem 5-ii?

Copy and paste relevant sections from the manuscript (include quotes in quotation marks "like this" to indicate direct quotes from your manuscript), or elaborate on this item by providing additional information not in the ms, or briefly explain why the item is not applicable/relevant for your study

"Our Spanish-language Web-based ¡Alégrate! positive psychological intervention was adapted from previously published and empirically validated curricula of Moskowitz's MARIGOLD trial [38-42]."

5-iii) Revisions and updating

Revisions and updating. Clearly mention the date and/or version number of the application/intervention (and comparator, if applicable) evaluated, or describe whether the intervention underwent major changes during the evaluation process, or whether the development and/or content was "frozen" during the trial. Describe dynamic components such as news feeds or changing content which may have an impact on the replicability of the intervention (for unexpected events see item 3b).

	1	2	3	4	5	
subitem not at all important	○	○	○	○	○	essential

Does your paper address subitem 5-iii?

Copy and paste relevant sections from the manuscript (include quotes in quotation marks "like this" to indicate direct quotes from your manuscript), or elaborate on this item by providing additional information not in the ms, or briefly explain why the item is not applicable/relevant for your study

Your answer

5-iv) Quality assurance methods

Provide information on quality assurance methods to ensure accuracy and quality of information provided [1], if applicable.

	1	2	3	4	5	
subitem not at all important	○	○	○	○	○	essential

Does your paper address subitem 5-iv?

Copy and paste relevant sections from the manuscript (include quotes in quotation marks "like this" to indicate direct quotes from your manuscript), or elaborate on this item by providing additional information not in the ms, or briefly explain why the item is not applicable/relevant for your study

Your answer

5-v) Ensure replicability by publishing the source code, and/or providing screenshots/screen-capture video, and/or providing flowcharts of the algorithms used

Ensure replicability by publishing the source code, and/or providing screenshots/screen-capture video, and/or providing flowcharts of the algorithms used. Replicability (i.e., other researchers should in principle be able to replicate the study) is a hallmark of scientific reporting.

	1	2	3	4	5	
subitem not at all important	○	○	○	○	○	essential

Does your paper address subitem 5-v?

Copy and paste relevant sections from the manuscript (include quotes in quotation marks "like this" to indicate direct quotes from your manuscript), or elaborate on this item by providing additional information not in the ms, or briefly explain why the item is not applicable/relevant for your study

Your answer

5-vi) Digital preservation

Digital preservation: Provide the URL of the application, but as the intervention is likely to change or disappear over the course of the years; also make sure the intervention is archived (Internet Archive, webcitation.org, and/or publishing the source code or screenshots/videos alongside the article). As pages behind login screens cannot be archived, consider creating demo pages which are accessible without login.

	1	2	3	4	5	
subitem not at all important	○	○	○	○	○	essential

Does your paper address subitem 5-vi?

Copy and paste relevant sections from the manuscript (include quotes in quotation marks "like this" to indicate direct quotes from your manuscript), or elaborate on this item by providing additional information not in the ms, or briefly explain why the item is not applicable/relevant for your study

"enrollees accessed content using the following URL: [https://www.marigoldstudy.org/alegrate/login/index.php]."

5-vii) Access

Access: Describe how participants accessed the application, in what setting/context, if they had to pay (or were paid) or not, whether they had to be a member of specific group. If known, describe how participants obtained "access to the platform and Internet" [1]. To ensure access for editors/reviewers/readers, consider to provide a "backdoor" login account or demo mode for reviewers/readers to explore the application (also important for archiving purposes, see vi).

	1	2	3	4	5	
subitem not at all important	○	○	○	○	○	essential

Does your paper address subitem 5-vii? *

Copy and paste relevant sections from the manuscript (include quotes in quotation marks "like this" to indicate direct quotes from your manuscript), or elaborate on this item by providing additional information not in the ms, or briefly explain why the item is not applicable/relevant for your study

"Each participant logs in to the ¡Alégrate! Web-site using a unique username and password assigned by research staff—and, the tablet interface is customized with shortcuts for ease of navigation."

5-viii) Mode of delivery, features/functionalities/components of the intervention and comparator, and the theoretical framework

Describe mode of delivery, features/functionalities/components of the intervention and comparator, and the theoretical framework [6] used to design them (instructional strategy [1], behaviour change techniques, persuasive features, etc., see e.g., [7, 8] for terminology). This includes an in-depth description of the content (including where it is coming from and who developed it) [1],” whether [and how] it is tailored to individual circumstances and allows users to track their progress and receive feedback” [6]. This also includes a description of communication delivery channels and – if computer-mediated communication is a component – whether communication was synchronous or asynchronous [6]. It also includes information on presentation strategies [1], including page design principles, average amount of text on pages, presence of hyperlinks to other resources, etc. [1].

	1	2	3	4	5	
subitem not at all important	○	○	○	○	◉	essential

Does your paper address subitem 5-viii? *

Copy and paste relevant sections from the manuscript (include quotes in quotation marks "like this" to indicate direct quotes from your manuscript), or elaborate on this item by providing additional information not in the ms, or briefly explain why the item is not applicable/relevant for your study

"Our 5-week ¡Alégrate! intervention delivers curricular content via didactic instruction (e.g., text, videos), journaling, and assigned at-home practice—all accessed online using investigator-purchased tablet computers. The website was designed and managed by Michael Cohn, a co-investigator of ¡Alégrate!. This five-session, multi-component Web-based intervention, instructs on eight emotion regulation skills which have proven efficacious for increasing positive emotion and decreasing symptoms of depression and psychological distress in patients with varying chronic illness diagnoses, i.e., those with metastatic breast cancer, HIV, and type 2 diabetes [39, 41, 42]. Detailed description of the curricula is published elsewhere [43]. Briefly, eight empirically validated behavioral and cognitive skills are taught via our Web-based interface, and include the following: (1) identification and use of personal strengths; (2) noticing of positive events in daily life; (3) prolonged appreciation and relishing of positive events; (4) positive reappraisal of stressful events or situations; (5) gratitude; (6) regular practice of mindfulness and meditation; (7) setting and working toward pragmatic and achievable goals; and (8) planning and performing acts of kindness. We have previously tested in-person delivery of MARIGOLD in the Spanish language [19]."

"Participants randomly assigned to the active control condition are asked to visit the study site where they report their emotions (e.g., grateful, happy, guilty, relieved, ashamed, etc.), thrice weekly, using the Differential Emotions Scale (DES), but—they are not granted access to curricular content of our ¡Alégrate! positive psychological intervention."

5-ix) Describe use parameters

Describe use parameters (e.g., intended “doses” and optimal timing for use). Clarify what instructions or recommendations were given to the user, e.g., regarding timing, frequency, heaviness of use, if any, or was the intervention used ad libitum.

	1	2	3	4	5	
subitem not at all important	○	○	○	○	○	essential

Does your paper address subitem 5-ix?

Copy and paste relevant sections from the manuscript (include quotes in quotation marks "like this" to indicate direct quotes from your manuscript), or elaborate on this item by providing additional information not in the ms, or briefly explain why the item is not applicable/relevant for your study

"As such, participants are instructed to login to the ¡Alégrate! Web-site for 30 minute sessions at least three time per week. New skills are sequentially taught every week (see Table 1 for sequencing and content); to ensure successive instruction, new content only becomes available after a 7-day period and once participants have completed the prior week’s content and at-home practice."

"It takes 5-10 minutes to complete the DES on-line."

5-x) Clarify the level of human involvement

Clarify the level of human involvement (care providers or health professionals, also technical assistance) in the e-intervention or as co-intervention (detail number and expertise of professionals involved, if any, as well as “type of assistance offered, the timing and frequency of the support, how it is initiated, and the medium by which the assistance is delivered”. It may be necessary to distinguish between the level of human involvement required for the trial, and the level of human involvement required for a routine application outside of a RCT setting (discuss under item 21 – generalizability).

	1	2	3	4	5	
subitem not at all important	○	○	○	○	○	essential

Does your paper address subitem 5-x?

Copy and paste relevant sections from the manuscript (include quotes in quotation marks "like this" to indicate direct quotes from your manuscript), or elaborate on this item by providing additional information not in the ms, or briefly explain why the item is not applicable/relevant for your study

"In addition, staff hold in-person 15-20 minute tutorials at baseline to familiarize enrollees on tablet use, with assurance of continual availability via phone or in-person to assist with any issues and to troubleshoot difficulties encountered with the technology."

5-xi) Report any prompts/reminders used

Report any prompts/reminders used: Clarify if there were prompts (letters, emails, phone calls, SMS) to use the application, what triggered them, frequency etc. It may be necessary to distinguish between the level of prompts/reminders required for the trial, and the level of prompts/reminders for a routine application outside of a RCT setting (discuss under item 21 – generalizability).

	1	2	3	4	5	
subitem not at all important	○	○	○	○	◉	essential

Does your paper address subitem 5-xi? *

Copy and paste relevant sections from the manuscript (include quotes in quotation marks "like this" to indicate direct quotes from your manuscript), or elaborate on this item by providing additional information not in the ms, or briefly explain why the item is not applicable/relevant for your study

"In addition to accessing our Web-based positive psychological intervention, participants also receive weekly phone calls and text messages to reinforce lessons learned and as a strategy to maximize retention and continued visitation to our study site. Weekly phone calls are brief, 10-15 minutes in duration, in which study staff review highlights of skills taught that week—along with tech support on any issues in handling the tablet computer or in navigating the ¡Alégrate! site. Finally, participants with cell phone capability receive a weekly text message from research staff highlighting the skills taught that week with the reminder to put them into daily practice—these, sometimes take the form of a 'meme' containing both images and text."

"Concerted efforts are undertaken to promote retention among participants in the control arm, including postcard notices and phone calls to remind participants to engage in on-line emotion reporting and to appear, in-person, for scheduled clinical assessments."

5-xii) Describe any co-interventions (incl. training/support)

Describe any co-interventions (incl. training/support): Clearly state any interventions that are provided in addition to the targeted eHealth intervention, as ehealth intervention may not be designed as stand-alone intervention. This includes training sessions and support [1]. It may be necessary to distinguish between the level of training required for the trial, and the level of training for a routine application outside of a RCT setting (discuss under item 21 – generalizability.

	1	2	3	4	5	
subitem not at all important	○	○	○	○	○	essential

Does your paper address subitem 5-xii? *

Copy and paste relevant sections from the manuscript (include quotes in quotation marks "like this" to indicate direct quotes from your manuscript), or elaborate on this item by providing additional information not in the ms, or briefly explain why the item is not applicable/relevant for your study

Not applicable; the pilot trial does not include any co-interventions.

6a) Completely defined pre-specified primary and secondary outcome measures, including how and when they were assessed

Does your paper address CONSORT subitem 6a? *

Copy and paste relevant sections from the manuscript (include quotes in quotation marks "like this" to indicate direct quotes from your manuscript), or elaborate on this item by providing additional information not in the ms, or briefly explain why the item is not applicable/relevant for your study

"Survey and clinical exams occur in tandem at three time points, as follows: Time 1 (baseline) at study enrollment; Time 2, immediately post-intervention (5-weeks after baseline); and Time 3 (12-weeks after baseline)."

"Measures of sitting and ambulatory blood pressure serve as the primary outcomes for the current ¡Alégrate! trial. An ambulatory BP monitoring (ABPM) method will be used to capture 24-hour daytime and nighttime BP readings in the natural environment (UltraliteTM 90217A ABPM from Spacelabs Healthcare); this device has shown adequate reliability and accuracy. We will consider 24-hour mean systolic and diastolic readings."

"We will additionally use an automatic sphygmomanometer to evaluate sitting BP. This measurement device has been validated across multiple cohort studies including MESA, NHANES, and HCHS/SOL. Three systolic and diastolic blood pressure readings will be taken with participants in the seated position; mean values will be obtained by averaging the last two readings."

"Specifically, secondary outcomes include: a) psychological well-being [depressive symptoms, perceived stress, positive and negative affect, optimism, emotional vitality, life engagement and meaning, happiness inducing behavior, and social support], (2) health behaviors [physical activity, diet, sodium intake, smoking status, sleep quality and duration, and mediation use], and (3) biological materials [serum blood spots]."

6a-i) Online questionnaires: describe if they were validated for online use and apply CHERRIES items to describe how the questionnaires were designed/deployed

If outcomes were obtained through online questionnaires, describe if they were validated for online use and apply CHERRIES items to describe how the questionnaires were designed/deployed [9].

	1	2	3	4	5	
subitem not at all important	○	○	○	○	○	essential

Does your paper address subitem 6a-i?

Copy and paste relevant sections from manuscript text

Not applicable; data will be collected during in-person interviews.

6a-ii) Describe whether and how “use” (including intensity of use/dosage) was defined/measured/monitored

Describe whether and how “use” (including intensity of use/dosage) was defined/measured/monitored (logins, logfile analysis, etc.). Use/adoption metrics are important process outcomes that should be reported in any ehealth trial.

	1	2	3	4	5	
subitem not at all important	○	○	○	○	○	essential

Does your paper address subitem 6a-ii?

Copy and paste relevant sections from manuscript text

"Finally, we will also analyze data describing overall use of the Web-site by trial participants, including the number of website logins per participant, average length of screen time per site visit, and extent to which enrollees completed and recorded practicing of at-home exercises."

6a-iii) Describe whether, how, and when qualitative feedback from participants was obtained

Describe whether, how, and when qualitative feedback from participants was obtained (e.g., through emails, feedback forms, interviews, focus groups).

	1	2	3	4	5	
subitem not at all important	○	○	○	○	○	essential

Does your paper address subitem 6a-iii?

Copy and paste relevant sections from manuscript text

"Additionally, we will conduct qualitative exit interviews to capture facilitators and barriers in using the tablet computer to access ¡Alégrate! curricular content. Indeed, qualitative process evaluation techniques further inform metrics of acceptability and utility for modality of content distribution."

6b) Any changes to trial outcomes after the trial commenced, with reasons

Does your paper address CONSORT subitem 6b? *

Copy and paste relevant sections from the manuscript (include quotes in quotation marks "like this" to indicate direct quotes from your manuscript), or elaborate on this item by providing additional information not in the ms, or briefly explain why the item is not applicable/relevant for your study

Not applicable; study is on-going and changes to trial outcomes have not been made.

7a) How sample size was determined

NPT: When applicable, details of whether and how the clustering by care provides or centers was addressed

7a-i) Describe whether and how expected attrition was taken into account when calculating the sample size

Describe whether and how expected attrition was taken into account when calculating the sample size.

	1	2	3	4	5	
subitem not at all important	○	○	○	○	○	essential

Does your paper address subitem 7a-i?

Copy and paste relevant sections from manuscript title (include quotes in quotation marks "like this" to indicate direct quotes from your manuscript), or elaborate on this item by providing additional information not in the ms, or briefly explain why the item is not applicable/relevant for your study

"We estimated the sample size requirements of a two-arm randomized trial to evaluate whether our Web-based positive psychological intervention results in differential and clinically meaningful improvements in systolic BP of 6 mmHg compared to an attention control condition. A repeated-measures design with systolic blood pressure determination at baseline, 5- and 12- weeks was used. In addition, the following operating characteristics were assumed: 1) a between-subject standard deviation for blood pressure of ; 2) a within-subject correlation, 3) a two-sided type I error probability of 0.05; 4) 80% statistical power. Frison and Pocock [65] argue that adjusting for baseline measures (ANCOVA) results in a more efficient analysis for a given sample size (larger statistical power). This approach yields an estimate of the required sample size of 28 participants per study arm under equal allocation. Furthermore, assuming a conservative attrition rate of 20% an updated estimation produces a sample size of participants per study arm. Stata 15 was used for sample size calculation (sampsi command) and all statistical analyses, with effect size estimation derived from published data on clinical effectiveness of psychotherapeutic interventions to treat hypertension [47, 66, 67]."

7b) When applicable, explanation of any interim analyses and stopping guidelines

Does your paper address CONSORT subitem 7b? *

Copy and paste relevant sections from the manuscript (include quotes in quotation marks "like this" to indicate direct quotes from your manuscript), or elaborate on this item by providing additional information not in the ms, or briefly explain why the item is not applicable/relevant for your study

"In a supplementary analysis, we will use ordinary or logistic regression (as appropriate, given variable distribution) to implement a dose-response analysis to test whether participation-related factors (e.g., number of sessions viewed, rate of homework completion) impact outcomes of interest."

[illegible]

8a) Method used to generate the random allocation sequence
NPT: When applicable, how care providers were allocated to each trial group

Does your paper address CONSORT subitem 8a? *

Copy and paste relevant sections from the manuscript (include quotes in quotation marks "like this" to indicate direct quotes from your manuscript), or elaborate on this item by providing additional information not in the ms, or briefly explain why the item is not applicable/relevant for your study

"The principal investigator created a random allocation table using Sealed Envelope, after which it was uploaded to REDCap to define the randomization model of ¡Alégrate! to ensure proper blocking and stratification by sex [male, female] and use of antihypertensive medication [yes, no]."

8b) Type of randomisation; details of any restriction (such as blocking and block size)

Does your paper address CONSORT subitem 8b? *

Copy and paste relevant sections from the manuscript (include quotes in quotation marks "like this" to indicate direct quotes from your manuscript), or elaborate on this item by providing additional information not in the ms, or briefly explain why the item is not applicable/relevant for your study

"After completing baseline assessments, participants are randomly assigned in a 1:1 ratio to the intervention or active control condition using block randomization alternating between varying block sizes of four, six, or eight."

9) Mechanism used to implement the random allocation sequence (such as sequentially numbered containers), describing any steps taken to conceal the sequence until interventions were assigned

Does your paper address CONSORT subitem 9? *

Copy and paste relevant sections from the manuscript (include quotes in quotation marks "like this" to indicate direct quotes from your manuscript), or elaborate on this item by providing additional information not in the ms, or briefly explain why the item is not applicable/relevant for your study

"A trained research staff generated allocation assignment through REDCap; note, REDCap conceals the allocation table and associated sequencing from research staff to prevent selection bias."

10) Who generated the random allocation sequence, who enrolled participants, and who assigned participants to interventions

Does your paper address CONSORT subitem 10? *

Copy and paste relevant sections from the manuscript (include quotes in quotation marks "like this" to indicate direct quotes from your manuscript), or elaborate on this item by providing additional information not in the ms, or briefly explain why the item is not applicable/relevant for your study

The principal investigator created a random allocation table using Sealed Envelope, after which it was uploaded to REDCap to define the randomization model of ¡Alégrate! to ensure proper blocking and stratification by sex [male, female] and use of antihypertensive medication [yes, no]. A trained research staff generated allocation assignment through REDCap; note, REDCap conceals the allocation table and associated sequencing from research staff to prevent selection bias. Finally, given the pilot nature of a Phase II trial, blinding occurs only at the level of data analysis.

11a) If done, who was blinded after assignment to interventions (for example, participants, care providers, those assessing outcomes) and how

NPT: Whether or not administering co-interventions were blinded to group assignment

11a-i) Specify who was blinded, and who wasn’t

Specify who was blinded, and who wasn’t. Usually, in web-based trials it is not possible to blind the participants [1, 3] (this should be clearly acknowledged), but it may be possible to blind outcome assessors, those doing data analysis or those administering co-interventions (if any).

	1	2	3	4	5	
subitem not at all important	○	○	○	○	○	essential

Does your paper address subitem 11a-i? *

Copy and paste relevant sections from the manuscript (include quotes in quotation marks "like this" to indicate direct quotes from your manuscript), or elaborate on this item by providing additional information not in the ms, or briefly explain why the item is not applicable/relevant for your study

"Finally, given the pilot nature of a Phase II trial, blinding occurs only at the level of data analysis."

11a-ii) Discuss e.g., whether participants knew which intervention was the “intervention of interest” and which one was the “comparator”

Informed consent procedures (4a-ii) can create biases and certain expectations - discuss e.g., whether participants knew which intervention was the “intervention of interest” and which one was the “comparator”.

	1	2	3	4	5	
subitem not at all important	○	○	○	○	○	essential

Does your paper address subitem 11a-ii?

Copy and paste relevant sections from the manuscript (include quotes in quotation marks "like this" to indicate direct quotes from your manuscript), or elaborate on this item by providing additional information not in the ms, or briefly explain why the item is not applicable/relevant for your study

"The consent form and study materials outline the design of the study and inform participants of random assignment into one of two groups, either the group where 'they learn skills to boost their mood and positive emotions' or the group where 'they would report their emotions on a thrice-weekly basis.'"

11b) If relevant, description of the similarity of interventions

(this item is usually not relevant for ehealth trials as it refers to similarity of a placebo or sham intervention to a active medication/intervention)

Does your paper address CONSORT subitem 11b? *

Copy and paste relevant sections from the manuscript (include quotes in quotation marks "like this" to indicate direct quotes from your manuscript), or elaborate on this item by providing additional information not in the ms, or briefly explain why the item is not applicable/relevant for your study

"Participants in the both the treatment and control arms receive detailed handouts on how to operate the Samsung Galaxy Tab A tablet computer, along with instructions on connecting to Wi-Fi and accessing the ¡Alégrate! web-site. Handouts display textual instructions along with detailed step-by-step visual screenshots, along with a listing of Wi-Fi locations in the local neighborhood, e.g., coffee shops and public libraries or bookstores."

12a) Statistical methods used to compare groups for primary and secondary outcomes

NPT: When applicable, details of whether and how the clustering by care providers or centers was addressed

Does your paper address CONSORT subitem 12a? *

Copy and paste relevant sections from the manuscript (include quotes in quotation marks "like this" to indicate direct quotes from your manuscript), or elaborate on this item by providing additional information not in the ms, or briefly explain why the item is not applicable/relevant for your study

"Descriptive statistics, at baseline, will summarize participant characteristics for the full sample and stratified by intervention arm. Bivariate tests will be conducted to compare the intervention and control conditions to ensure compatibility across key variables (e.g., demographic factors, well-being profiles, etc.) and to test success of the randomization protocol."

"Independent samples t-tests will be performed using 5-week systolic BP and diastolic BP as the dependent variables with treatment condition serving as the grouping variable. Other model-based approaches to handle missing data, such as weighted estimating equations, will be implemented. We will also use mixed effects models to compare changes in BP between the control and intervention arms as measured at baseline, 5 weeks, and 12 weeks. The independent variables include a time variable t (t = 0, 5, 12), a dummy variable N (N=1 if PP intervention group; N=0 if attention control), and a cross-product term (t X N). For ambulatory data collected across a 24-hour period, sensitivity analyses will compare mean overall daytime versus nighttime values of BP. All data analysis will be conducted using SAS 9.4 software (SAS Institute, Cary, NC)."

"Similar analytic techniques will be implemented for Aim 2 as used for Aim 1. The interaction of Group x Time will test if greater improvements are evident for psychological well-being and engagement in healthful behaviors (e.g., dietary intake and self-reported physical activity) at 5-weeks post-baseline for the treatment arm as compared to the active control group. RM-ANOVA will also be used to examine whether the '¡Alégrate!' intervention is associated with greater improvements in psychological well-being and engagement in healthful behavior. In addition to reporting nominal p-values, we will document the number of tests conducted and associated Bonferroni correction. Finally, we will conduct mediation analysis to test whether psychological well-being and health behaviors serve as intermediates (or mediators) through which ¡Alégrate! impacts blood pressure control. Tests of mediation will follow recommendations of Shrout and Bolder to bootstrap product terms using Mplus. Given dependency of indirect effects on the time interval, for multiwave models we will compute overall, rather than time-specific, indirect effects."

"Similar analytic techniques as previously described will be used when testing intervention effects on chronic inflammation. Specifically, the term capturing Group x Time interaction will test whether lower levels of inflammation are evident at 8-weeks post-baseline for the intervention group vs. the active control arm."

12a-i) Imputation techniques to deal with attrition / missing values

Imputation techniques to deal with attrition / missing values: Not all participants will use the intervention/comparator as intended and attrition is typically high in ehealth trials. Specify how participants who did not use the application or dropped out from the trial were treated in the statistical analysis (a complete case analysis is strongly discouraged, and simple imputation techniques such as LOCF may also be problematic [4]).

	1	2	3	4	5	
subitem not at all important	○	○	○	○	◉	essential

Does your paper address subitem 12a-i? *

Copy and paste relevant sections from the manuscript (include quotes in quotation marks "like this" to indicate direct quotes from your manuscript), or elaborate on this item by providing additional information not in the ms, or briefly explain why the item is not applicable/relevant for your study

"Additionally, we will document the number of missing observations over time to assess potential for bias resulting from significant dropout and differential attrition by condition. We will perform independent sample t-tests and/or Fisher's exact tests to compare baseline characteristics of enrollees who completed the trial versus those who prematurely dropped out, allowing us to test possible predictors of attrition."

"Sensitivity analysis will use multiple imputation procedures across missing values to ensure inclusion of all observations, particularly those resulting from participants who withdraw, are lost to follow-up, or do not complete all assessments."

"Independent samples t-tests will be performed using 5-week systolic BP and diastolic BP as the dependent variables with treatment condition serving as the grouping variable. Other model-based approaches to handle missing data, such as weighted estimating equations, will be implemented."

12b) Methods for additional analyses, such as subgroup analyses and adjusted analyses

Does your paper address CONSORT subitem 12b? *

Copy and paste relevant sections from the manuscript (include quotes in quotation marks "like this" to indicate direct quotes from your manuscript), or elaborate on this item by providing additional information not in the ms, or briefly explain why the item is not applicable/relevant for your study

"In a supplementary analysis, we will use ordinary or logistic regression (as appropriate, given variable distribution) to implement a dose-response analysis to test whether participation-related factors (e.g., number of sessions viewed, rate of homework completion) impact outcomes of interest."

X26) REB/IRB Approval and Ethical Considerations [recommended as subheading under "Methods"] (not a CONSORT item)

X26-i) Comment on ethics committee approval

	1	2	3	4	5	
subitem not at all important	○	○	○	○	○	essential

Does your paper address subitem X26-i?

Copy and paste relevant sections from the manuscript (include quotes in quotation marks "like this" to indicate direct quotes from your manuscript), or elaborate on this item by providing additional information not in the ms, or briefly explain why the item is not applicable/relevant for your study

"The Institutional Review Board at the University of Illinois at Urbana-Champaign (UIUC) approved the ¡Alégrate! trial and all subjects gave written informed consent."

x26-ii) Outline informed consent procedures

Outline informed consent procedures e.g., if consent was obtained offline or online (how? Checkbox, etc.?), and what information was provided (see 4a-ii). See [6] for some items to be included in informed consent documents.

	1	2	3	4	5	
subitem not at all important	○	○	○	○	○	essential

Does your paper address subitem X26-ii?

Copy and paste relevant sections from the manuscript (include quotes in quotation marks "like this" to indicate direct quotes from your manuscript), or elaborate on this item by providing additional information not in the ms, or briefly explain why the item is not applicable/relevant for your study

"The consent form and study materials outline the design of the study and inform participants of random assignment into one of two groups, either the group where “they learn skills to boost their mood and positive emotions” or the group where “they would report their emotions on a thrice-weekly basis.” It is communicated that all curricular instruction occurs on-line by visiting our ¡Alégrate! website. Additionally, staff inform participants that three in-person clinical visits are required, each 2-3 hour in length, where staff collect survey and clinical data, including collection of anthropometric information, blood spots, and 24-hour blood pressure monitoring; home visits are also offered for data collection to increase rates of retention. Participants are informed that they keep the investigator-purchased tablet computer at the end of the study as compensation for their participation and time invested [37]."

"Participants expressing interest in enrolling in the trial who are screened for full eligibility and ultimately qualify, undergo a face-to-face consent process and complete the survey and clinical exams prior to randomization."

X26-iii) Safety and security procedures

Safety and security procedures, incl. privacy considerations, and any steps taken to reduce the likelihood or detection of harm (e.g., education and training, availability of a hotline)

	1	2	3	4	5	
subitem not at all important	○	○	○	○	○	essential

Does your paper address subitem X26-iii?

Copy and paste relevant sections from the manuscript (include quotes in quotation marks "like this" to indicate direct quotes from your manuscript), or elaborate on this item by providing additional information not in the ms, or briefly explain why the item is not applicable/relevant for your study

"Note, all data will be de-identified, stored in a password protected computer, and/or will be stored in a locked cabinet in the principal investigator’s office."

"Each participant logs in to the ¡Alégrate! Web-site using a unique username and password assigned by research staff—and, the tablet interface is customized with shortcuts for ease of navigation. Participants are also instructed to password protect their tablet to mitigate any data breaches from outside parties."

RESULTS

13a) For each group, the numbers of participants who were randomly assigned, received intended treatment, and were analysed for the primary outcome

NPT: The number of care providers or centers performing the intervention in each group and the number of patients treated by each care provider in each center

Does your paper address CONSORT subitem 13a? *

Copy and paste relevant sections from the manuscript (include quotes in quotation marks "like this" to indicate direct quotes from your manuscript), or elaborate on this item by providing additional information not in the ms, or briefly explain why the item is not applicable/relevant for your study

Not applicable; data collection is on-going.

13b) For each group, losses and exclusions after randomisation, together with reasons

Does your paper address CONSORT subitem 13b? (NOTE: Preferably, this is shown in a CONSORT flow diagram) *

Copy and paste relevant sections from the manuscript (include quotes in quotation marks "like this" to indicate direct quotes from your manuscript), or elaborate on this item by providing additional information not in the ms, or briefly explain why the item is not applicable/relevant for your study

Not applicable; data collection is on-going.

13b-i) Attrition diagram

Strongly recommended: An attrition diagram (e.g., proportion of participants still logging in or using the intervention/comparator in each group plotted over time, similar to a survival curve) or other figures or tables demonstrating usage/dose/engagement.

	1	2	3	4	5	
subitem not at all important	○	○	○	○	○	essential

Does your paper address subitem 13b-i?

Copy and paste relevant sections from the manuscript or cite the figure number if applicable (include quotes in quotation marks "like this" to indicate direct quotes from your manuscript), or elaborate on this item by providing additional information not in the ms, or briefly explain why the item is not applicable/relevant for your study

Not applicable; data collection is on-going.

14a) Dates defining the periods of recruitment and follow-up

Does your paper address CONSORT subitem 14a? *

Copy and paste relevant sections from the manuscript (include quotes in quotation marks "like this" to indicate direct quotes from your manuscript), or elaborate on this item by providing additional information not in the ms, or briefly explain why the item is not applicable/relevant for your study

"Activities of the Spanish-language ¡Alégrate! intervention were funded in 2017, and data collection is on-going. We expect to submit trial results for peer-reviewed publication in 2021, soon after recruitment has concluded and statistical analysis are finalized."

14a-i) Indicate if critical “secular events” fell into the study period

Indicate if critical “secular events” fell into the study period, e.g., significant changes in Internet resources available or “changes in computer hardware or Internet delivery resources”

	1	2	3	4	5	
subitem not at all important	○	○	○	○	○	essential

Does your paper address subitem 14a-i?

Copy and paste relevant sections from the manuscript (include quotes in quotation marks "like this" to indicate direct quotes from your manuscript), or elaborate on this item by providing additional information not in the ms, or briefly explain why the item is not applicable/relevant for your study

Not applicable; recruitment is on-going and no need to change computer hardware or delivery resources has emerged.

14b) Why the trial ended or was stopped (early)

Does your paper address CONSORT subitem 14b? *

Copy and paste relevant sections from the manuscript (include quotes in quotation marks "like this" to indicate direct quotes from your manuscript), or elaborate on this item by providing additional information not in the ms, or briefly explain why the item is not applicable/relevant for your study

Not applicable; data collection is on-going.

15) A table showing baseline demographic and clinical characteristics for each group

NPT: When applicable, a description of care providers (case volume, qualification, expertise, etc.) and centers (volume) in each group

Does your paper address CONSORT subitem 15? *

Copy and paste relevant sections from the manuscript (include quotes in quotation marks "like this" to indicate direct quotes from your manuscript), or elaborate on this item by providing additional information not in the ms, or briefly explain why the item is not applicable/relevant for your study

Not applicable; data collection is on-going.

15-i) Report demographics associated with digital divide issues

In ehealth trials it is particularly important to report demographics associated with digital divide issues, such as age, education, gender, social-economic status, computer/Internet/ehealth literacy of the participants, if known.

	1	2	3	4	5	
subitem not at all important	○	○	○	○	○	essential

Does your paper address subitem 15-i? *

Copy and paste relevant sections from the manuscript (include quotes in quotation marks "like this" to indicate direct quotes from your manuscript), or elaborate on this item by providing additional information not in the ms, or briefly explain why the item is not applicable/relevant for your study

Not applicable; data collection is on-going.

16) For each group, number of participants (denominator) included in each analysis and whether the analysis was by original assigned groups

16-i) Report multiple “denominators” and provide definitions

Report multiple “denominators” and provide definitions: Report N’s (and effect sizes) “across a range of study participation [and use] thresholds” [1], e.g., N exposed, N consented, N used more than x times, N used more than y weeks, N participants “used” the intervention/comparator at specific pre-defined time points of interest (in absolute and relative numbers per group). Always clearly define “use” of the intervention.

	1	2	3	4	5	
subitem not at all important	○	○	○	○	○	essential

Does your paper address subitem 16-i? *

Copy and paste relevant sections from the manuscript (include quotes in quotation marks "like this" to indicate direct quotes from your manuscript), or elaborate on this item by providing additional information not in the ms, or briefly explain why the item is not applicable/relevant for your study

"The independent variables include a time variable t (t = 0, 5, 12), a dummy variable N (N=1 if PP intervention group; N=0 if attention control), and a cross-product term (t X N)."

16-ii) Primary analysis should be intent-to-treat

Primary analysis should be intent-to-treat, secondary analyses could include comparing only "users", with the appropriate caveats that this is no longer a randomized sample (see 18-i).

	1	2	3	4	5	
subitem not at all important	○	○	○	○	○	essential

Does your paper address subitem 16-ii?

Copy and paste relevant sections from the manuscript (include quotes in quotation marks "like this" to indicate direct quotes from your manuscript), or elaborate on this item by providing additional information not in the ms, or briefly explain why the item is not applicable/relevant for your study

"Given our design and main objective of treatment evaluation, an intent-to-treat analysis will be conducted where we will consider data of all participants randomized—with retention of their original intervention assignment."

17a) For each primary and secondary outcome, results for each group, and the estimated effect size and its precision (such as 95% confidence interval)

Does your paper address CONSORT subitem 17a? *

Copy and paste relevant sections from the manuscript (include quotes in quotation marks "like this" to indicate direct quotes from your manuscript), or elaborate on this item by providing additional information not in the ms, or briefly explain why the item is not applicable/relevant for your study

Not applicable; data collection is on-going.

17a-i) Presentation of process outcomes such as metrics of use and intensity of use

In addition to primary/secondary (clinical) outcomes, the presentation of process outcomes such as metrics of use and intensity of use (dose, exposure) and their operational definitions is critical. This does not only refer to metrics of attrition (13-b) (often a binary variable), but also to more continuous exposure metrics such as “average session length”. These must be accompanied by a technical description how a metric like a “session” is defined (e.g., timeout after idle time) [1] (report under item 6a).

	1	2	3	4	5	
subitem not at all important	○	○	○	○	○	essential

Does your paper address subitem 17a-i?

Copy and paste relevant sections from the manuscript (include quotes in quotation marks "like this" to indicate direct quotes from your manuscript), or elaborate on this item by providing additional information not in the ms, or briefly explain why the item is not applicable/relevant for your study

Not applicable; data is on-going.

17b) For binary outcomes, presentation of both absolute and relative effect sizes is recommended

Does your paper address CONSORT subitem 17b? *

Copy and paste relevant sections from the manuscript (include quotes in quotation marks "like this" to indicate direct quotes from your manuscript), or elaborate on this item by providing additional information not in the ms, or briefly explain why the item is not applicable/relevant for your study

Not applicable; data collection is on-going.

18) Results of any other analyses performed, including subgroup analyses and adjusted analyses, distinguishing pre-specified from exploratory

Does your paper address CONSORT subitem 18? *

Copy and paste relevant sections from the manuscript (include quotes in quotation marks "like this" to indicate direct quotes from your manuscript), or elaborate on this item by providing additional information not in the ms, or briefly explain why the item is not applicable/relevant for your study

Not applicable; data collection is on-going.

18-i) Subgroup analysis of comparing only users

A subgroup analysis of comparing only users is not uncommon in ehealth trials, but if done, it must be stressed that this is a self-selected sample and no longer an unbiased sample from a randomized trial (see 16-iii).

	1	2	3	4	5	
subitem not at all important	○	○	○	○	○	essential

Does your paper address subitem 18-i?

Copy and paste relevant sections from the manuscript (include quotes in quotation marks "like this" to indicate direct quotes from your manuscript), or elaborate on this item by providing additional information not in the ms, or briefly explain why the item is not applicable/relevant for your study

Not applicable; data collection is on-going.

19) All important harms or unintended effects in each group

(for specific guidance see CONSORT for harms)

Does your paper address CONSORT subitem 19? *

Copy and paste relevant sections from the manuscript (include quotes in quotation marks "like this" to indicate direct quotes from your manuscript), or elaborate on this item by providing additional information not in the ms, or briefly explain why the item is not applicable/relevant for your study

"Note, all data will be de-identified, stored in a password protected computer, and/or will be stored in a locked cabinet in the principal investigator's office."

"Each participant logs in to the ¡Alégrate! Web-site using a unique username and password assigned by research staff—and, the tablet interface is customized with shortcuts for ease of navigation. Participants are also instructed to password protect their tablet to mitigate any data breaches from outside parties."

19-i) Include privacy breaches, technical problems

Include privacy breaches, technical problems. This does not only include physical "harm" to participants, but also incidents such as perceived or real privacy breaches [1], technical problems, and other unexpected/unintended incidents. "Unintended effects" also includes unintended positive effects [2].

	1	2	3	4	5	
subitem not at all important	○	○	○	○	○	essential

Does your paper address subitem 19-i?

Copy and paste relevant sections from the manuscript (include quotes in quotation marks "like this" to indicate direct quotes from your manuscript), or elaborate on this item by providing additional information not in the ms, or briefly explain why the item is not applicable/relevant for your study

"Each participant logs in to the ¡Alégrate! Web-site using a unique username and password assigned by research staff—and, the tablet interface is customized with shortcuts for ease of navigation. Participants are also instructed to password protect their tablet to mitigate any data breaches from outside parties."

19-ii) Include qualitative feedback from participants or observations from staff/researchers

Include qualitative feedback from participants or observations from staff/researchers, if available, on strengths and shortcomings of the application, especially if they point to unintended/unexpected effects or uses. This includes (if available) reasons for why people did or did not use the application as intended by the developers.

	1	2	3	4	5	
subitem not at all important	○	○	○	○	○	essential

Does your paper address subitem 19-ii?

Copy and paste relevant sections from the manuscript (include quotes in quotation marks "like this" to indicate direct quotes from your manuscript), or elaborate on this item by providing additional information not in the ms, or briefly explain why the item is not applicable/relevant for your study

Not applicable; data collection is on-going.

DISCUSSION

22) Interpretation consistent with results, balancing benefits and harms, and considering other relevant evidence

NPT: In addition, take into account the choice of the comparator, lack of or partial blinding, and unequal expertise of care providers or centers in each group

22-i) Restate study questions and summarize the answers suggested by the data, starting with primary outcomes and process outcomes (use)

Restate study questions and summarize the answers suggested by the data, starting with primary outcomes and process outcomes (use).

	1	2	3	4	5	
subitem not at all important	○	○	○	○	○	essential

Does your paper address subitem 22-i? *

Copy and paste relevant sections from the manuscript (include quotes in quotation marks "like this" to indicate direct quotes from your manuscript), or elaborate on this item by providing additional information not in the ms, or briefly explain why the item is not applicable/relevant for your study

Not applicable; data collection is on-going.

22-ii) Highlight unanswered new questions, suggest future research

Highlight unanswered new questions, suggest future research.

	1	2	3	4	5	
subitem not at all important	○	○	○	○	○	essential

Does your paper address subitem 22-ii?

Copy and paste relevant sections from the manuscript (include quotes in quotation marks "like this" to indicate direct quotes from your manuscript), or elaborate on this item by providing additional information not in the ms, or briefly explain why the item is not applicable/relevant for your study

"Future trials may want to explore more user-friendly modalities that require little to no coaching that may be more appealing to less technological savvy community members."

"Future trials may want to deploy a more active control condition where participant's access Web-based content unrelated to the outcome of interest, e.g., money management skills."

"How might technology be deployed within a more user-friendly interface for those with limited exposure to technology or those with little formal educational training who experience literacy challenges?"

20) Trial limitations, addressing sources of potential bias, imprecision, and, if relevant, multiplicity of analyses

20-i) Typical limitations in ehealth trials

Typical limitations in ehealth trials: Participants in ehealth trials are rarely blinded. Ehealth trials often look at a multiplicity of outcomes, increasing risk for a Type I error. Discuss biases due to non-use of the intervention/usability issues, biases through informed consent procedures, unexpected events.

	1	2	3	4	5	
subitem not at all important	○	○	○	○	○	essential

Does your paper address subitem 20-i? *

Copy and paste relevant sections from the manuscript (include quotes in quotation marks "like this" to indicate direct quotes from your manuscript), or elaborate on this item by providing additional information not in the ms, or briefly explain why the item is not applicable/relevant for your study

"Selective enrollment has the potential to introduce bias as Hispanics/Latinos with limited technological proficiency may decline to participate in the trial, or if enrolled, may have difficulty in connecting to Wi-Fi or in navigating the study site."

"We are recruiting in a Chicago-based neighborhood with a large populace of Hispanics/Latino adults, the majority of which are of Mexican ancestry. Thus, findings of the trial may not generalize to the wider more heterogeneous Hispanic/Latino population across the U.S. with differences by country of origin, nativity status, and level of acculturation."

"Finally, blinding occurs only at the level of data analysis and both trial participants and assessors (research staff collecting outcome data) are aware of randomization assignment, which can introduce unintended bias."

21) Generalisability (external validity, applicability) of the trial findings

NPT: External validity of the trial findings according to the intervention, comparators, patients, and care providers or centers involved in the trial

21-i) Generalizability to other populations

Generalizability to other populations: In particular, discuss generalizability to a general Internet population, outside of a RCT setting, and general patient population, including applicability of the study results for other organizations

	1	2	3	4	5	
subitem not at all important	○	○	○	○	○	essential

Does your paper address subitem 21-i?

Copy and paste relevant sections from the manuscript (include quotes in quotation marks "like this" to indicate direct quotes from your manuscript), or elaborate on this item by providing additional information not in the ms, or briefly explain why the item is not applicable/relevant for your study

"We are recruiting in a Chicago-based neighborhood with a large populace of Hispanics/Latino adults, the majority of which are of Mexican ancestry. Thus, findings of the trial may not generalize to the wider more heterogeneous Hispanic/Latino population across the U.S. with differences by country of origin, nativity status, and level of acculturation."

21-ii) Discuss if there were elements in the RCT that would be different in a routine application setting

Discuss if there were elements in the RCT that would be different in a routine application setting (e.g., prompts/reminders, more human involvement, training sessions or other co-interventions) and what impact the omission of these elements could have on use, adoption, or outcomes if the intervention is applied outside of a RCT setting.

	1	2	3	4	5	
subitem not at all important	○	○	○	○	○	essential

Does your paper address subitem 21-ii?

Copy and paste relevant sections from the manuscript (include quotes in quotation marks "like this" to indicate direct quotes from your manuscript), or elaborate on this item by providing additional information not in the ms, or briefly explain why the item is not applicable/relevant for your study

"Finally, we provided all enrollees with an investigator-purchased tablet computer and we developed detailed Spanish-language user guides (with screenshots) to assist participants in accessing our website—with, research staff available via phone for additional tech support."

OTHER INFORMATION

OTHER INFORMATION

23) Registration number and name of trial registry

Does your paper address CONSORT subitem 23? *

Copy and paste relevant sections from the manuscript (include quotes in quotation marks "like this" to indicate direct quotes from your manuscript), or elaborate on this item by providing additional information not in the ms, or briefly explain why the item is not applicable/relevant for your study

"This work was funded by the Rosenfeld Heart Foundation and is registered at clinicaltrials.gov under registration number NCT03892057."

24) Where the full trial protocol can be accessed, if available

Does your paper address CONSORT subitem 24? *

Cite a Multimedia Appendix, other reference, or copy and paste relevant sections from the manuscript (include quotes in quotation marks "like this" to indicate direct quotes from your manuscript), or elaborate on this item by providing additional information not in the ms, or briefly explain why the item is not applicable/relevant for your study

Description of the full trial protocol can be accessed at clinicaltrials.gov under registration number NCT03892057.

25) Sources of funding and other support (such as supply of drugs), role of funders

Does your paper address CONSORT subitem 25? *

Copy and paste relevant sections from the manuscript (include quotes in quotation marks "like this" to indicate direct quotes from your manuscript), or elaborate on this item by providing additional information not in the ms, or briefly explain why the item is not applicable/relevant for your study

"Additionally, research reported in this publication was supported, in part, by the National Heart, Lung, and Blood Institute under award number 1K01HL130712 and the National Institute on Minority Health and Health Disparities of the National Institutes of Health (NIH) under award number U54MD012523."

X27) Conflicts of Interest (not a CONSORT item)

X27-i) State the relation of the study team towards the system being evaluated

In addition to the usual declaration of interests (financial or otherwise), also state the relation of the study team towards the system being evaluated, i.e., state if the authors/evaluators are distinct from or identical with the developers/sponsors of the intervention.

	1	2	3	4	5	
subitem not at all important	○	○	○	○	○	essential

Does your paper address subitem X27-i?

Copy and paste relevant sections from the manuscript (include quotes in quotation marks "like this" to indicate direct quotes from your manuscript), or elaborate on this item by providing additional information not in the ms, or briefly explain why the item is not applicable/relevant for your study

"The authors have no conflicts of interest in this work. None declared."

About the CONSORT EHEALTH checklist

As a result of using this checklist, did you make changes in your manuscript? *

- ○ yes, major changes
- ◉ yes, minor changes
- ○ no

What were the most important changes you made as a result of using this checklist?

Your answer

How much time did you spend on going through the checklist INCLUDING making changes in your manuscript *

8 hours

As a result of using this checklist, do you think your manuscript has improved? *

- ◉ yes
- ○ no
- ○ Other:

Would you like to become involved in the CONSORT EHEALTH group?

This would involve for example becoming involved in participating in a workshop and writing an "Explanation and Elaboration" document

- ○ yes
- ◉ no
- ○ Other:

Any other comments or questions on CONSORT EHEALTH

Your answer

STOP - Save this form as PDF before you click submit

To generate a record that you filled in this form, we recommend to generate a PDF of this page (on a Mac, simply select "print" and then select "print as PDF") before you submit it.

When you submit your (revised) paper to JMIR, please upload the PDF as supplementary file.

Don't worry if some text in the textboxes is cut off, as we still have the complete information in our database. Thank you!

Final step: Click submit !

Click submit so we have your answers in our database!

Submit

Never submit passwords through Google Forms.

This content is neither created nor endorsed by Google. Report Abuse - Terms of Service - Privacy Policy

Google Forms
